# Economic Feasibility Study of a Carbon Capture and Storage (CCS) Integration Project in an Oil-Driven Economy: The Case of the State of Kuwait

**DOI:** 10.3390/ijerph19116490

**Published:** 2022-05-26

**Authors:** Adel Naseeb, Ashraf Ramadan, Sultan Majed Al-Salem

**Affiliations:** 1 Techno-Economics Department, Science and Technology Sector, Kuwait Institute for Scientific Research (KISR), P.O. Box 24885, Safat 13109, Kuwait; ajnaseeb@kisr.edu.kw; 2 Environment and Life Sciences Research Centre, Kuwait Institute for Scientific Research (KISR), P.O. Box 24885, Safat 13109, Kuwait; aramadan@kisr.edu.kw

**Keywords:** levelized cost of electricity, CCS, heavy oil, Kuwait, NPV, IRR

## Abstract

The rapid growth and urbanization rate, coupled with hot climate and scarce rainfall, makes it essential for a country like Kuwait to have several power and desalination plants with high-generating capacity. These plants are entirely reliant on burning fossil fuels as a source of thermal energy. These plants are also universally accepted to be the largest CO_2_ emitters; hence, they present a potential for carbon capture and storage (CCS). Having established the suitability of the existing conditions for post-combustion CCS, a techno-economic-based feasibility study, which took into consideration local power generation technologies and economic conditions, was performed. Relying on fifteen case study models and utilizing the concept of levelized cost of electricity (LCOE), the statistical average method (SAM) was used to assess CCS based on realistic and reliable economic indicators. Zour power station, offering the highest potential CO_2_ stream, was selected as a good candidate for the analysis at hand. Heavy fuel oil (HFO) was assumed to be the only fuel type used at this station with affixed price of USD 20/barrel. The analysis shows that the internal rate of return (IRR) was about 7%, which could be attributed to fuel prices in Kuwait and governmental support, i.e., waived construction tax and subsidized workforce salaries. Furthermore, the net present value (NPV) was also estimated as USD 47,928 million with a 13-year payback period (PBP). Moreover, 1–3% reductions in the annual operational cost were reflected in increasing the IRR and the NPV to 9–11% and USD 104,085–193,945 million, respectively, and decreasing the PBP to 12–11 years. On the contrary, increasing the annual operational cost by 1% made the project economically unfeasible, while an increase of 3% resulted in negative IRR (−1%), NVP (−USD 185,458 million) and increased PBP to 30 years. Similarly, increasing the HFO barrel price by USD 5 resulted in negative IRR (−10%) and NVP (−USD 590,409); hence, a CCS project was deemed economically unfeasible. While the study considered the conditions in Kuwait, it is expected that similar results could be obtained for other countries with an oil-driven economy. Considering that around 62% of the fossil fuel blend in Kuwait is consumed by electricity and water generation, it is inevitable to consider the possibility and practicality of having a carbon network with neighboring countries where other oil-driven economies, such as Kingdom of Saudi Arabia and Iraq, can utilize a CCS-based mega infrastructure in Kuwait. The choice of Kuwait is also logical due to being a mid-point between both countries and can initiate a trading scheme in oil derivatives with both countries.

## 1. Introduction

The recently published report of Working Group III of the Intergovernmental Panel on Climate Change (IPCC) still sets a core concept to reach net-zero carbon dioxide (CO_2_) emissions when anthropogenic carbon emissions are balanced globally by anthropogenic CO_2_ removals [1]. This is achieved by diversifying energy baskets, reducing existing carbon emissions from various industrial sources, and aligning efforts to reach the set target of having a 77% increase in electricity production from renewable energy feedstock. There are also various schemes set in place to regulate carbon emissions from various anthropogenic sources, such as the European Commission’s (EC) emission trading system (ETS) with measurable targets till the year 2050 [2]. Various countries within the developed world have also committed themselves to measurable targets to keep global warming temperatures well below the 2 °C mark by signing the Paris Agreement on Climate Change [3]. To achieve such goals, especially considering existing industrial infrastructure for developed and developing countries alike, carbon capture and storage (CCS) offers itself as solution to mitigate carbon emission strength from various sectors. Presently, there are 74 operational CCS projects which have successfully utilized different technologies and 43 are under construction due to go into operation as of late 2018 [4,5]. It is also well established that the largest CO_2_ emitter around the globe is the power sector, namely utilizing coal, natural gas, and fuel oil as a source to its gas/steam turbines with an average emission strength 3.94, 0.77–1.01, and 0.55–1.27 MtCO_2_ per source [6]. Table S1 in the Supplementary Materials depicts the total estimated emissions around the globe from the power sector and the number of each source by fuel type. The reader is also referred to Table S2 for other anthropogenic sources by type and emission strength around the globe. We bring the kind attention of the reader that the letter S used prior to the number of figures and tables stands for material presented in the Supplementary Materials presented electronically to compliment this article.

CCS technologies vary significantly in terms of their capture techniques used. In fact, not all carbon streams are appropriate for capture technologies and the logistics for storing the carbon on- or off-site also present a logistical burden. The typical carbon stream captured should have a partial pressure of 0.1 MPa and a CO_2_ concentration (by vol.%) of 3 to 4% [7]. Table S3 shows the different carbon streams that could be captured from various streams within a power plant and their properties. In addition, CCS technologies are summarized in Table S4, depicting the main commercial technologies of market maturity and the principle behind each technique. It should be noted that based on the review conducted and depicted in the aforementioned table, amine absorption in post-combustion technology was noted to be the most mature and popular amongst other options. Although studies within new materials and configurations used in CCS have been published as of late, there seems to be a gap in literature concerning the economic feasibility of projects, such as CCS, namely utilized for power stations which are considered to be the main carbon emitter amongst industrial sources. Furthermore, literature is quite scant for feasibility studies considering regions with an altered economy in Europe or China, such as oil-driven ones where prices vary along with fuel and infrastructure. In a fairly recent study by Koelbl et al. [8], two scenarios were studied in detail using a trade-linked model to analyze the upstream economic behavior for the Netherlands. These scenarios were (i) an 80% emission reduction scheme by 2050 in comparison to the 1990 carbon levels; and (ii) a renewable energy basket strategy for the power sector. It was concluded that the CCS-exclusive scenario for the power sector was less favorable for gross value added (GVA) and energy security when compared to a renewable basket of energy with a biomass focus. Nonetheless, it should be noted that such scenarios consider various imports from outside country boarders and favor nuclear power (consequently uranium) imports [9]. In addition, CCS, in the case of the Netherlands as a non-oil dependent economy, may also become dependent on natural gas and fuel imports for the power sector. Al-Qayim et al. [10] have evaluated the techno-economic performance of biomass fuel for combustion power plants, both with and without CCS in the UK, with different coal used as a fuel. Two CCS technologies were applied, resulting in different performances. On the other hand, using biomass has produced negative-emissions of carbon in general. Oxy-fuel CC resulted in a 14% higher efficiency and 6% lower cost of electricity (COE) than post-combustion CC. In order to boost biomass CCS, a critical price of GBP 55.2 per ton was projected in the study. Fan et al. [11] has demonstrated that retrofitting CCS projects did not achieve the optimal investment value till the year 2027, when compared with renewable strategies in China and after studying total investment value (TIV) with respect to each scenario’s net present value (NPV). On the other hand, Xianzheng et al. [12] studied a model of wind power and coal fired power plant to analyze their economic performance. The NPV decreased along with the COE and carbon avoided cost with wind power, indicating worse financial performance in Northwest China. In this work, we consider the largest power plant within the State of Kuwait and conduct a detailed feasibility study for a CCS project that utilizes post-combustion as a technology to capture the carbon being emitted. The work is quite unique based on the fact that it carries detailed analysis in an oil-driven economy with various advantageous options to have such projects commissioned. This is due to their economic and environmental benefits. The article is divided into a number of sections to ease the flow of technical information for the reader. It starts with a general introduction section, detailing the main theme of the research work and a background/literature survey. The section that follows shows the detailed methodology carried out after describing the main characteristics of the country and power plant stations considered. The methods section includes the carbon capture and storage technology selection process, the market size and drivers of cost analysis, as well as economic and sensitivity analysis. After which, a dedicated section is presented to detail the results and discuss its relevance, whilst final conclusions are drawn from the work and presented in a separate section towards the end.

## 2. Methods

### 2.1. Country Description and Power Sector’s Carbon Strength

The State of Kuwait is located on the Western Arabian Peninsula (29°30′ N lat. And 47°45′ E long.), noted to be one of the highest countries in the world in terms of gross domestic product (GDP) [13]. It is also an oil-dependent nation that is a member of both the Organization of the Petroleum Exporting Countries (OPEC) and the Organization of Arab Petroleum Exporting Countries (OAPEC), and ranks within the top ten countries of crude oil production and its reserves. The power and energy sector is operated mainly using fossil fuels derived from crude oil downstream activities within its borders. Past efforts in terms of quantifying and identifying carbon emissions and sources in the State of Kuwait with the aim of its future capture and sequestration are quite minimal [14,15,16,17,18,19]. These studies concluded a number of essential technical points: (i) that enhanced oil recovery (EOR) is not possible in Kuwait due to logistical reasons, scattering of sources, and dilution of carbon concentrations; and that (ii) the highest source of carbon is the power sector [20,21,22]. A full-carbon Atlas has also been published for Kuwait, quantifying all carbon sources to date [18]. There are also no regulations declared by governing bodies with regards to CCS in Kuwait, and it should also be noted at this stage that the recent International Energy Agency (IEA) reports show that Kuwait, on average, consumes more primary energy (577 BTU per Capita) than the world (75 BTU per Capita), Europe (134 BTU per Capita), and the Middle East (142 BTU per capita) [23,24]. This shows that the associated carbon footprint of Kuwait, with regards to its various carbon sources previously discussed in Al-Salem [19] and Al-Mutairi et al. [18], is somewhat justified since all of such sources are dependent on combusting crude oil derivatives and emit CO_2_ to the atmosphere.

**Figure 1:**
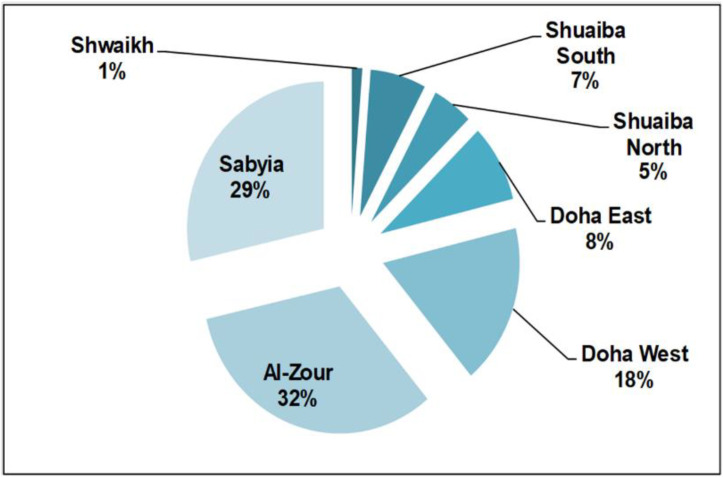
Breakdown (percentile) of power stations in Kuwait by carbon strength.

The power sector is owned and managed by the Ministry of Electricity and Water (MEW), which on the other hand subsidizes the electricity prices for residents to (USD 0.016 per kWh) as of the year 2017 [25]. This shows that the average resident pays some 5% of the actual utility price. Furthermore, all power stations in Kuwait are operated using fossil fuels and are combined with water desalination units. Both climatic conditions (e.g., reliance on air conditioning for cooling) and rapid population growth (3.3%) are considered to be the main influencing factors for high energy demand in Kuwait [18,20]. The IPCC has set various guidelines and methods to estimate carbon emissions, namely from the power sector. These methods are appropriate for stationary sources and could be summarized as a reference approach, a sectoral approach (Tier 1), and a bottom-up approach (Tier 2) [6]. The latter is considered to be the most common and reliable approach as it relies on technology assessment of end-user based on the different scenarios considered. There are seven power stations in Kuwait that consume natural gas, heavy and gas oil, and heavy crude, and its derivatives constitute a share of 82% of the total consumption [26]. Other than the major concern of burning crude oil in power stations to compensate high demand in summer months, thermal steam turbines make up 85% of the ones used for operation, which are also associated with higher carbon emissions when compared to gas turbines [27]. The power stations, as well as their declared capacity and primary fuel used, are shown in Table S5. A breakdown of each station by capacity of each turbine type is also shown in Table S6 for the reader’s consideration. A previous assessment for Kuwait based on the IPCC Tier 2 approach shows that the 41.636 MtCO_2_ y-1 is emitted from the power sector using the emission factors (EFs) depicted in Table S6 for each turbine/station. The power sector is responsible for 41.6% of total carbon strength of the country and a breakdown by station is drawn for the reader’s consideration and shown in Figure 1 along with total fuel consumed in Table S7. Further details could be found elsewhere in Al-Mutairi et al. [18]. An assessment study based on the method used and a sensitivity analysis is also carried out and illustrated in Annex in the Supplementary Materials using the latest version of the IPCC inventory software (i.e., V2.54; IPCC, Geneva, Switzerland). This is carried out to provide a comprehensive overview of the case of Kuwait for each power station considered.

### 2.2. Economic Analysis, Process Evaluation, and Technical Considerations

The objective of this work is to demonstrate the viability on an economic basis for CCS in an oil-driven market. As previously shown in Table S5, there are seven power stations in Kuwait with a total installed capacity of around 18 thousand MW. The smallest PS is Shuwaikh with (250 MW); additionally, the largest PS is Al-Zour with installed capacity of 5306.7 MW. Furthermore, the average installed capacity of all stations is around 2200 MW of electricity, which is very similar to station Doha West, in terms of production capacity. Therefore, Doha West is presented as a viable and ideal candidate to represent a stream of CO_2_ suitable for CCS based on average capacity [28]. Al-Zour is also considered in the first stages of assessment as the largest station in terms of capacity. The fuel consumed and capacity are shown in Table S7.

#### 2.2.1. Carbon Capture and Storage Technology Selection

Several CCS technologies are available for deployment around the world. However, some factors can affect the selection process of the CCS technology, such as the CO_2_ concentration in the gas streams, technology maturity, and fuel types. Accordingly, there are three CCS systems suitable for implementation in the State of Kuwait post-combustion, pre-combustion, and oxy-combustion [6,29]. Pre-combustion techniques can capture the carbon stream after separating the syngas stream post-reforming (Table S4). In other words, the pre-combustion system processes the fuel with pure oxygen or air steam to produce synthesis gas or syngas. Moreover, the key components of this syntheses gas/syngas are carbon monoxide (CO) and hydrogen gas (H_2_). Although this system is ideal for separating high concentrations of CO_2_ in gas streams, the processes are considered to be elaborate and costly in power stations [6]. Oxy-combustion systems require large amounts of energy to complete the separation process. Additionally, once the process of oxy-combustion system is finalized, two new components are generated (water and CO_2_), which are added to the separation costs and storage. In technical terms, post-combustion systems capture the CO_2_ from flue gases that are produced by combustion of fossil fuels. Additionally, the remaining fuel gas is passed through a separation process, by injecting liquid or solid absorbents which can capture the CO_2_. Therefore, the CO_2_ can be injected into an oil reservoir (RO) for storage, and the remaining flue gas is discharged to the atmosphere. Furthermore, this system is considered to be economically feasible due to several factors, such as technological similarities with the power stations and market maturity; thus, it is selected for the case at hand.

#### 2.2.2. Market Size and Cost Drivers

Around the globe, China has the most substantial electricity demand, reaching up to 5320 terawatt per hour (TWh). Additionally, in the projected year of 2040, electricity demand is estimated to increase by 3910 TWh and to reach around 9230 TWh. Furthermore, the second largest country in electricity demand is the United States, reaching approximately 3886 TWh in 2016, and is projected to reach about 4570 TWh in 2040 [30]. Locally within Kuwait, the government provides energy and desalinated water to households, as well as commercial and industrial sectors. Table 1 illustrates the State of Kuwait demand and supply of power as declared by the state. Moreover, the production of electricity in 2016 is augmented to reach 18,870 MW. Additionally, these increments in electricity over the past few decades are attributable to several factors, such as population growth, economic prosperity, and technological innovations. Table 2 below shows the global and local prices of each fuel source utilized in the State of Kuwait which are considered in this work [31]. The cost of HO is between USD 20–38 per barrel internationally, and locally it is around USD 35 per barrel. Furthermore, the natural gas (NG) prices locally and globally are very similar at USD 3.55 and USD 3 million British thermal unit (MMBTU), respectively. Additionally, the international price of CO is around USD 51 per barrel, while locally is around USD 40 per barrel. Correspondingly, the prices of gas oil (GO) internationally and domestically are both at around USD 53 per barrel. Therefore, this comparison process indicates that MEW fuel costs behavior is driven by international markets prices (Table 2).

#### 2.2.3. Pricing Method and Carbon Market

There are various factors that affect the cost of electricity, such as fuel prices fluctuating, different power generation technologies and their efficiencies, and power transmission losses. Therefore, a more stable cost calculation method is required to unify the different cost approaches used in the power generation (PG) industry [34]. Consequently, a levelized cost of electricity (LCOE) is developed in this work, consisting of an average revenue received per unit of the energy’s output, for the first year of operation with an annual rate of null (zero). In other words, the LCOE can be defined either by the energy price of electricity or by the net present value (NPV) with a zero investment rate [35]. NPV analysis is chosen as it presents a number of advantages, namely accounting for the future depreciation of investment, the possibility of future investment return analysis, and the fact that it can take account of actual value assets (such as capital costs). The LCOE of different conventional PG teleology’s price ranges, which can be utilized for the recommended technologies and adapted from Lazard [33]. Furthermore, the CO_2_ market is divided into several segments, including oil and gas industries, food and beverage industries, medical industries, and fire-fighting industries [36]. Additionally, the worldwide demand of CO_2_ is around 80 Mtpa, and the major share of the market is enhanced oil recovery (EOR), representing 63% [37]. In addition, the current price of CO_2_ is around USD 40 per ton of CO_2_ equivalent—an estimate set by the market’s economical behavior. Furthermore, it is expected that this current price may increase in the near future and reach USD 80 per ton of CO_2_ equivalent. Therefore, the growth of the carbon market, especially in the Middle East, allows governments to invest into technologies such as CCS for the purpose of cost reduction or revenue generation [36].

### 2.3. Techno-Economic Assessment and Evaluation

**Figure 2:**
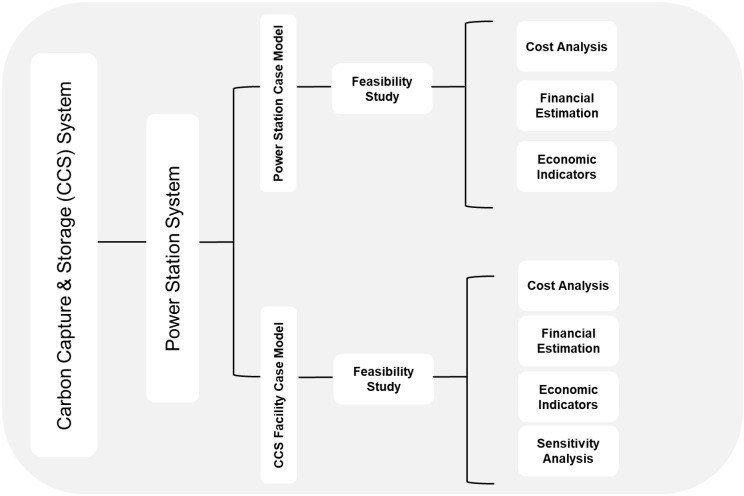
Methodology followed to conduct feasibility study based on techno-economic evaluation.

In order to conduct a feasibility study based on a techno-economic evaluation, three prior consecutive steps that could be treated as individual studies must be completed by selecting a CCS system, a PS system, and the case models, as depicted in Figure 2, showing the tree structure for the methodology followed. The CCS system is chosen by applying the statistical average method on the low-cost accounting technique (SAMLCAT). In other words, multiple case study models (CSMs) of the selected systems mentioned for the CCS are presented, and the system with the lowest average cost (LAC) is chosen as the primary system. Therefore, the assessment is conducted on sound grounds, and the power station and the CCS project based on economic indicators is evaluated, resulting in the most realistic evaluation, as encompassed in Figure 2. Moreover, in the second step, a power station system is selected, based on the power generation technologies and economic conditions that are applicable for MEW in the State of Kuwait. Additionally, in the third consecutive step, a CSM is selected for two entities—the power station installed capacity (PSIC) and the CCS facility (CCSF)—based on the literature review and statistical average methods (Figure 2) [38]. Furthermore, and once completed, the feasibility study is used to evaluate the above selected CSMs. Moreover, and as shown in the tree methodology depicted, there are two feasibility studies—one for the power station case model (PSCM) and the other for the CCSF case model (CCSFCM). Additionally, the first feasibility study has three sections—cost analysis, financial estimation, and economic indicators. Similarly, the second feasibility study contains four sections—cost analysis, financial estimation, economic indicators, and sensitivity analyses for the CCS project.

Additionally, the process of SAMLCAT depends on fifteen CSMs, distributed equally between the systems. Furthermore, once the calculation process of SAMLCAT is completed, the LAC method is selected for this feasibility study [31]. A selection process is instigated for the appropriate power technology that can be adapted with the suggested CCS technology [32].

To select the superlative PSCM that can be applied to its related feasibility study, two processes are used—the PSIC selection process and the PSCM selection process. Accordingly, the PSIC selection process is based upon a statistical average method of Kuwait’s PSIC’s in the year 2016 [18]. By way of explanation, the average PSIC is calculated from Table S6, and consequently the selection process method is based on the nearest Kuwait’s PSIC to the calculated average PSIC. Additionally, in the second process of this section, several CSMs are presented from the literature review. Moreover, each CSM contains a financial statement (FLS), displaying the cost of construction and operation of PS with CCSF. Furthermore, the total cost (TC) of each FLS in its related CSM is collected and arranged, as shown in Table 3. Furthermore, the average cost (AC) of the arranged TCs is calculated; therefore, the FLS that contains the nearest TC value to the AC is selected as the basis for its FS. In addition, the FLS structure of the selected CSM is based on the methodology previously depicted [31].

### 2.4. Carbon Capture and Storage (CCS) Facility Case Model

The TC of the selected FLS related to CM is evaluated, and the AC calculation method is initiated. Moreover, the TC with the nearest value to the AC is chosen; therefore, its FLS acts as the basis, as shown in Table 4 [38].

#### 2.4.1. Cost Estimation

The foundation of the feasibility method is based on determining the appropriate TC and the cost of electricity (COE) of the PSCM and the CCSFCM. These are conducted on the basis of following costs combined:

- Raw materials: This cost sums the annual raw materials required to construct, operate, and produce the finished product.
- Equipment: This cost group consists of the annual cost of equipment required to construct, operate, and produce the finished product.
- Labor: This cost group encompasses the annual administrative and operational labor cost for constructing, operating, and producing the finished product.
- Utilities: This cost group covers the annual services cost, such as electricity, water, and oil associated with constructing, operating, and producing the finished product.
- Buildings and structures: This cost group includes the annual costs for building and structures that are required to construct, operate, and produce the finished product.
- Fixed assets: This cost group consists of the cost of the above two cost groups, as well as the building equipment and structures, which are used to construct the essence of the power plant and the CCSF.
- Amortization and depreciation: This cost group involves the depreciation calculation cost for the fixed assets that are utilized in the construction and operation of the power plant and CCSF.
- Total cost: This cost group includes the aggreged totals of all of the above cost groups, as well as other costs that can affect the outcome of the final product. Additionally, the above costs groups are then restructured and reorganized based on the total cost methodology of (TCM).
- Cost of electricity (COE): Once all of the above are calculated, the final cost is estimated for COE, as per the following [42].

(1) COE=(TOC+OCFIX+OCVAR)Annual Net MWh Generated=Total CostTotal Prodcution

where TOC stands for the first-year capital costs (charges) (USD), OCFIX is the first-year operating costs (USD), and OCVAR is the first-year variable operating costs (USD) (see Tables S10–S18).

#### 2.4.2. Financial Estimation

In order to estimate the financial costing for the power generated in the power plant, as per the followed methodology, four previously mentioned components are calculated as per the following (Figure 1).

- Pre-production cost: To construct the power plant or CCSF and monitor the construction process, an administrative headquarters setup is required. This cost group, due to calculation process position significance, is aligned to this financial group. Furthermore, the components of this financial group are shown in Table S19.
- Revenue: This is the first financial group that is considered as the basis of the other groups. This category is the result of a multiplication process between price and quantity, as shown [31,43].

(2) Revenue=Price×Prodcution Quantity (PQ)

A variation of the above formula is derived to be applicable in this feasibility study; therefore, the new revenue formula is a multiplication process between LOCE, representing the price, and the power plant output (PPO), representing the quantity of production.

(3) Revenue=LOCE×PPO

- Working capital: The objective of this financial group is to calculate the required amount for the purpose of operating the project. In addition, this financial group consists of three main cost groups (salaries, raw materials, and utilities), as shown and estimated in Table S18.
- Initial investment: This is the third financial group which includes a mixture of cost and financial groups to allow in the construction and operation of the project, as illustrated in Table S19.
- Net cash flow: This is the final financial group which calculates the net cash flow (NCF) of the project for the duration of its life span, taken as 30 years. In other words, the purpose of this financial group is to calculate the profits or losses, as depicted below.

NCF = Cash-In − Cash-Out(4)

Additionally, in the adjusted version of the formula above, the cash-in is replaced by revenue and cash-out is replaced by operational cost, as shown in the formula below [44].

NCF = Revenue − Operational Cost(5)

#### 2.4.3. Economic Indicators

The economic indicators considered in the feasibility study are summarized below and are aimed at determining the profitability of the project after setting up the initial investment costs above. To estimate the net present Value (*NPV*) in today’s money, the formula below is used [45].

(6) NPV=∑t(year)=1nNCFt(1+r)t−CF0

Furthermore, the internal rate of return (*IRR*) is estimated as it represents the compound interest of the present values for the in-flow (revenue), equaling the current value of cash out-flow (expenses). By way of explanation, the *IRR* is the present value of in-flows equaling the investment value when *NPV* is zero [45,46].

(7) IRR=∑t(year)=1nCFt(1+IRR)t−CF0

As for the payback period (*PBP*), which signifies the period or length of time required to return the capital investment of the project, it is estimated as the following [45]. The return on investment (*ROI*) is also subsequently calculated [47].

(8) $$PBP=\frac{Investment\;Value}{\sum NCF}$$

(9) $$ROI=\frac{NPV}{Intial\;Investment}\times 100$$

## 3. Results and Discussion

In this section, we present the results of the CSM related to the power station and the CCSF. The selection process for the PSCM and the CCSM is based on the calculations performed using the SAMLCAT and the AC. An adjustment is also performed by replacing the PSIC in the FLS to fit the case at hand for the State of Kuwait. Moreover, the fuel source in FLS is replaced with the Kuwaiti heavy oil (HO) fuel source (Table S7). Furthermore, the consumption capacity is changed, depending on the selected Kuwait’s PS-HO consumption capacity, leading to a change in the annual fuel cost variable. The IGGC-PS is the most compatible technology for the sustainability of the CCSF, based on the available information allocated from MEW and the reviewed literature. Moreover, the calculated AC amount from the applied statistical method is around USD 2.5 billion, based on the results depicted in detail within Tables S9–S18. According to the study conducted by the NETL [48], the cost is within the average of CSM for such a project. Hence, to accommodate the largest plant with potential carbon stream appropriate for CCS, we select the Al-Zour station with an installed capacity of 5805.80 MW annually so that the integrated plant can consider its facility. Additionally, the selected fuel source for this FLS is HO, with an average price of USD 34 per barrel, which is very similar to MEW cost (Table S20). Moreover, the selected LCOE price considered is USD/MW 175, and the T and S cost is 5% from TC [48].

### 3.1. CCSF Selection Process

The objective of this section is to select the CSM for the CCSF applied in the integrated plan for the power station (Al-Zour). By way of explanation, the expected life span of power stations is around 30–35 years (Table S14). This surpasses stations available in Kuwait within the MEW. Therefore, an assumption is made to eliminate the construction cost of a power plant with a CCSF and calculate the TC of a CCSF for an existing MEW-PS instead, which are adapted and calculated based on the data extracted from NTEL [35]. The calculated AC of the above CSMs is around USD 303 million, which is in line with previous findings (NTEL, 2001). It should also be kept in mind that both feasibility studies in this work aim to assess the power station and the CCS project integrated with it, which have an investment rate of 6% [20].

### 3.2. Cost Analysis

The first cost group to be estimated is the raw materials (RMs) which amounts to USD 93 million. The second cost group is the equipment cost required to construct and operate the station instead of a retrofitting project which sums to around USD 821 million since the station surpasses its life expectancy and requires more money to maintain it, rather than constructing a new one with modified specifications and applicability for CCS (Tables S9 and S10). Additionally, the third group in this category is labor, where its annual cost is used to construct and operate the station (USD 323 million). Utility costs subsidized at the governmental rate is the fourth cost group, amounting to roughly USD 3.5 thousand. Fixed assets, which sum the two cost groups amounting to USD 840 million, is also considered (Table S14). Furthermore, depreciation and amortization also strictly show the depreciation of the total assets over the project’s life span (i.e., 30 years) (Table S15). The TC is estimated at USD 905 million in the first production year, as shown in Table S16. Additionally, this amount fluctuates over the years and stabilizes in year twelve at USD 885,712 million over the remaining life span of the project. Table 5 shows the summary of the cost outputs considered for the Al-Zour power station which is also applicable for constructing a new station to substitute the old one, instead of retrofitting the existing power plant and desalination plant in Kuwait. The CCS project can accommodate 1.7 Mtpa of CO_2_ produced from the station, with an amine absorption-based unit (see variation of carbon calculation in Annex in the Supplementary Materials).

### 3.3. Financial Estimation

The pre-production cost is assessed to accommodate the cost requirements of the administration buildings. The total amount that is required to construct and operate the headquarters is around USD 53 million, as per the calculated categories depicted in Table S16. The initial investment is estimated as USD 840 million, and the pre-production and the working capital costs amount to USD 420 and USD 53 million, respectively. Therefore, the total amount of this financial group is around USD 1.3 billion. The revenues from the power station are estimated with a LCOE of 175 USD/MW. The feasibility study associated with the power station yields USD 1 billion; an annual breakdown is given in Table S22. The LCOE is sensitive to various variables, such as the plant capacity, capital investment, and fuel cost [49]. These are investigated and discussed in the sensitivity analysis section, whilst the capacity is kept fixed to accommodate the carbon captured in the considered station (e.g., Al-Zour). Additionally, in the first few years of operations, the NCF project is worth around USD 135 million (Table S21). Furthermore, the NCF increases slightly over the years to reach an amount of USD 144 million. Moreover, the NCF in year twelve stabilizes at USD 187 million for the remaining production year. As for the economic indicators related to the constructed CCS project working on an amine absorption principle, Table 6 summarizes the feasibility study results for the project. Therefore, the COE of the CCSF is around 168 USD/MW. Furthermore, in comparing the feasibilities both COEs, it is realized that the COE-PS is much lower than COE-CCSF; in fact, it is even lower than the LCOE selected for this feasibility. Moreover, one of the reasons for difference in the COE values is due to the PSIC in both FSs.

As for the economic indicators for the CCS project, the IRR is estimated to be 7%, presenting a lucrative ratio for such projects. This can be attributed to the fuel prices in Kuwait and, moreover, the government subsidies for such projects, which are considered in this work, such as elimination construction tax, supporting labor salaries. The NPV is also estimated as USD 47,928 million, and the PBP is estimated as 13 years and 1 month with an ROI equaling at 20%.

### 3.4. Sensitivity Analysis for CCS Project

The sensitivity analysis presented in this section is aimed at showing the reliance of this project against changes anticipated within the work and the local market. A baseline scenario is chosen for the LCEO at 175 USD/MW, PSIC at 2541 MW, and HO (Kuwaiti crude), with a price of USD 20 per barrel representing the lower end of the price spectrum when compared with average oil prices. Table 7 illustrates seven hypothetical calculated cost adjustment scenarios for the CCSF-FS. Additionally, the first scenario in the middle is the base study which displays the economic indicators aforementioned. Moreover, the remaining six scenarios are divided into two segments—three with an increase in operational cost and three with a decrease in the same category, which represents the group that could be most influenced by changes in the market. Therefore, in the first scenario, for the above, the operational cost is in the decreasing segment, reaching 1% over the life span of the project. Accordingly, the IRR shows a profitable ratio at 9%, and NPV at USD 104,085 million, which signifies a valuable approach for such projects in Kuwait. Furthermore, the PBP is 12 years and the ROI is 44%, demonstrating a beneficial financial outcome as well. Consequently, in the second and third scenarios of the operational costs, the reduced ratios are 2% and 3%, respectively, leading to an IRR of 10% and 11, correspondingly. Likewise, the NPV of both scenarios is USD 152,327 million and USD 193,945 million, respectively, while the PBP for both scenarios is 11 years. Therefore, the above operational cost reduction scenarios show a favorable financial outcome. Additionally, in the second segment of the scenarios, the operational costs are increased to show a different outcome. However, in the first scenario, the operational cost is increased by 1%, which leads to a significant decrease in the economic indicators. Moreover, in the first indicator, the IRR is reduced to 5%, and the NPV is also decreased, reaching USD 17,713 million. Additionally, the PBP is 15 years and the ROI is −7%; therefore, this scenario economic indicators all display the unfeasibility of the project (highlighted in Table 7). Furthermore, in the second scenario, the operational cost increases by 2%, leading to a 1% decrease in the IRR, thus indicating an unfeasible project. In addition, the NPV, PBP, and ROI are all reduced to reach negative USD 94,738 million across 19 years, i.e., a −40% decrease, respectively. Correspondingly, in the third scenario, the operational cost increases by 3%, which results in a negative IRR, thus indicating unfeasible outcomes of the projects. Moreover, the remaining economic indicators of this scenario present an unfeasible result concerning the project. Therefore, any increase in the operational cost for the base study scenario can lead to a significant decrease in the project’s profitability outcome.

Table 8 illustrates the price adjustment scenarios in the HO for the CCSF-FS, containing three base study scenarios (business as usual baseline), including A-15 and A-25. In the A-15 scenario, the price of HO is decreased to USD 15 per barrel, while the price of HO increases to USD 25 per barrel in the A-25 scenario. In addition, in the A-15 scenario, the IRR is 41%, the NPV is USD 1164 billion, and the PBP is 2 years, while the ROI is a very high ratio of 487%, indicating a highly feasible project, as shown in Table 8. Additionally, in the second scenario, the price of HO increases up to 25 USD/barrel, which is very similar to its cost in MEW. Therefore, by applying this price, an increase in HO in this scenario can result in adverse economic outcomes. The first economic indicator is IRR, showing a negative 10%; likewise, the NPV shows a negative value of USD 590,409 million. Additionally, the PBP displays a period over 30 years, and the ROI has an unfavorable ratio of −447%. Therefore, any increase in the HO prices beyond the baseline can result in an unfeasible project, as demonstrated. It should be noted that the results may demonstrate a certain tendency to showcase a non-feasible project with any increase expected in the local market for crude oil prices (fuel derivatives), which might lead to neglecting CCS projects in an economy, such as Kuwait. However, the background must be discussed and considered here in detail to deliver a realistic viewpoint. There is a key limitation in conducting sensitivity scenarios for such work, where the focus is on constructing and operating a CCS plant devoted for the power sector as a standalone carbon emitter in Kuwait. In reality, the assumption that the CCS can withstand Al-Zour’s plant carbon emission and can also accommodate additional carbon streams from nearby refineries, namely the new refinery project in the same area [19]. Conducting sensitivity analysis on HO prices can exclusively neglect other impacts on various sectors in Kuwait which contribute to carbon sources. The exclusion of energy-saving measures and carbon crediting is also another limitation here and is also indicated in other previous studies [8]. In order to avoid an increase in COE and alterations in IRR with the HO process, carbon crediting schemes can take effect in Kuwait when implemented by governmental bodies, such as the Kuwait Environment Public Authority (KEPA). An allowance proved to be highly useful in the previous study of Bellotti et al. [50], in order to avoid COE increases.

As previously pointed, the bulk of carbon emissions in Kuwait are associated with the power sector essentially yielded from combusting fossil fuels. The commonly used fuel types in MEW P&D plants are gas oil, lean gas, fuel gas, and crude, depending on type of turbines. This might be of concern for future mitigation plans, where lesser carbon intense sources should be considered as turbines relying on natural gas rather than HO. The four most applicable technologies for CO_2_ capture from P&D plants include post-combustion, pre-combustion, oxy-fuel combustion, and chemical looping, which are also reviewed for the benefit of this study. Post-combustion is chosen as a mature choice in the market; however, the study might be extended to assess the feasibility of operating more than one choice in the future or more than one station to overcome logistical issues in storage and transport. Futuristic scenarios of direct extraction from air are also a possibility, namely for integration purposes with a CCS project in Kuwait, such as those presented in this study. This is essential in order to overcome the impracticality of extracting ambient CO_2_ concentrations (i.e., ~400 ppm), which are not feasible and require additional operating expenses [51].

From a macro point of view considering the region as a whole, the majority of the power generation in Middle East utilizes oil-fired power and desalination plants. Sixty percent of the world desalination capacity exists in the Middle East, particularly in the Gulf Cooperation Council (GCC) countries [52]. In the GCC countries, about 58% of the energy consumption is attributed to air conditioning and water desalination [23]. In Kuwait, over 80% of the blend of fossil-fuel-based primary energy is consumed via thermal conversion processes. Around total 62% of this amount is consumed by both electricity and water generation (≈52% for electricity production) and about (≈10%) for desalination freshwater production. This poses a direct question of possibility and practicality of having a carbon network with neighboring countries where other oil-driven economies, such as Saudi and Iraq, can utilize a CCS-based mega infrastructure in Kuwait. The choice of Kuwait is also logical due to being a mid-point between both countries and can initiate a trading scheme in oil derivatives with both countries. It should be noted herein that there is a policy gap in terms of most effective CCS technology and implementation policy in Kuwait, where no clear and published targets are put in place to accommodate the country’s status. It is quite common in developed nations to tax fossil fuel energy sources as one of the most common policy options to mitigate and control carbon emissions [53]. However, in Kuwait, where the sole means of energy is fossil-fuel-based, this seems to be a hindering step that requires proper infrastructure for renewables (e.g., waste to energy, solar, wind, etc.) and such projects are yet still not in their right stature to produce what is expected of them for the national grid. Therefore, this stage of country’s development requires more incentives for energy projects that can utilize renewable sources in order to reduce carbon emissions. Furthermore, judging from the baseline scenario (IRR of 7%), the right choice of CCS technology can be a lucrative option that can become economically self-sustaining with time. The emission issue can be solved with time and can be reduced as the project takes place in other parts of the world, as described by Duan et al. [53]. An internal policy of governmental based carbon taxing seems to be a viable step as well, especially on the oil and power sectors where such a policy implementation can definitely set a true course for action for commissioning CCS plants. KEPA is a regulatory body within the state similar to other parts of the world where a governmental body is responsible for monitoring the emissions of various sectors. With time, large-scale CCS may require policy implementation to accommodate large-scale plants, as well as initiatives to foster entrepreneurial activity and market formation, which can also extend to neighboring countries as well based on our study [54]. Such policies should also accommodate a no cap policy for storage in a similar fashion to the *Q45* policy for carbon credits [55]. This is used to accommodate the case of oil-driven economies such as Kuwait at initial stages of technology implementation. There are also a number of conclusions that can be drawn based on the empirical analysis conducted in this work, namely a comparative assessment with other Middle Eastern countries. As of the year 2022, the Middle East is in the lowest region in terms of CCS projects [56]. This should not be confused with the shear amount of carbon captured, as the region has 11 CCS operational projects located in Saudi, Qatar, and the Emirates, which are responsible for 10% of global CO_2_ captured annually (capacity extending 3.8 Mt per annum in some cases), i.e., double that of Europe. The focus of these projects is associated with gas processing plants (≈26% captured globally, 42.6 Mt per annum). Compared to Kuwait, which has no CCS plants as of yet, a good start may be to concentrate efforts on associated gas plants with the oil sector, as described elsewhere [19,20]. However, the power sector remains the largest in Kuwait as a prime emitter of carbo, as depicted earlier in this work (41.636 Mt per annum, 41.6%), which can also provide a good start for such CCS future plans. This is especially true when considering capital scaling and electrical power subsidies provided in the Middle East region, as previously depicted by Paltsev et al. [57]. Currently, the Emirates are ranked first in CCS projects in the region [58]. A carbon network can also offer a good start in Gulf countries between Kuwait, Saudi, and the Emirates.

During the years 2020–2021, the global COVID-19 pandemic has drastically altered energy policies around the world. This was due to a number of factors, namely general lockdowns which, on the other hand, reduced transportation and demand on fuels following boarder closures between countries. Daily carbon emissions reduced by 17%, as of early April 2020, when compared to the average of 2019 [59]. It should be noted that the drop in CO_2_ emissions had no detectable impact on atmospheric CO_2_ or climate change [60]. Therefore, it is estimated that CCS projects and future plans can recover as the Russian–Ukrainian conflict drives oil markets to new highs and the original demand shows to gain strength similar to before the pandemic. As of late, the issue of air quality, namely over the past three years, has been a focus of many health agencies where relevant. This is due to proven health effects of major pollutants on the general wellbeing of populations around the globe. Based on the transport sector data, Huang et al. [61] proposed a novel nonlinear multivariate grey model (ENGM (1,4)) based on an environmental Kuznets curve. This new tool was validated against the results from the US, Japan, and China, and during the years 2019–2025, an average increase in carbon emissions from the transport sector in China and the USA was 2.837% and 2.394%, respectively. The results of Japan show a downward trend with an average decline rate of 1.2231%. Therefore, the transport sector in mega populated cities (with largely dense countries) could benefit from such models to estimate and subsequently monitor their carbon emissions. The same principle could also be applied on different sectors and could be transferred to oil-driven economies, such as Kuwait. These sectors can also include industry [62], new emerging transport technologies [63], and pollution during the past pandemic [64].

## 4. Conclusions

The results have a certain tendency to showcase a non-feasible project with any increase expected in the local market for crude oil prices (fuel derivatives), which might lead to neglecting CCS projects in an economy such as Kuwait. However, there is a key limitation in conducting sensitivity scenarios for such work, where the focus is on constructing and operating a CCS plant devoted for power sectors as a standalone carbon emitter in Kuwait. In reality, there is an assumption that the CCS can withstand the carbon emissions of Al-Zour’s plant and can also accommodate additional carbon streams from nearby refineries (Kuwait’s fourth refinery is in the proximity of Al-Zour’s plant). Furthermore, conducting sensitivity analysis on HO prices exclusively neglects other impacts on various sectors in Kuwait that contribute to carbon sources. The exclusion of energy-saving measures and carbon crediting is also another limitation here, as indicated by other previous studies. In order to avoid an increase in COE and alterations in IRR with the HO process, carbon crediting schemes might take effect in Kuwait when implemented by governmental bodies, e.g., Kuwait Environment Public Authority (KEPA). As previously pointed, the bulk of carbon emissions in Kuwait are associated with the power sector essentially yielded from combusting fossil fuels. The commonly used fuel types in MEW P&D plants are gas oil, lean gas, fuel gas, and crude, depending on the type of turbines. This may present a concern for future mitigation plans where lesser carbon intense sources should be considered, as turbines rely on natural gas rather than HO. Post-combustion CCS is chosen as a mature choice in the market; however, the study might be extended to assess the feasibility of operating more than one choice in the future or more than one station to overcome logistical issues in storage and transport. Futuristic scenarios of direct extraction from air are also a possibility, namely for integration purposes with a CCS project in Kuwait, such as those depicted in this study. Considering that around 62% of the fossil fuel blend in Kuwait is consumed by electricity and water generation, it is inevitable to consider the possibility and practicality of having a carbon network with neighboring countries where other oil-driven economies, such as the Kingdom of Saudi Arabia and Iraq, can utilize a CCS-based mega infrastructure in Kuwait. The choice of Kuwait is also logical due to being a mid-point between both countries and can initiate a trading scheme in oil derivatives with both countries.

## Figures and Tables

**Table 1:** State of Kuwait power generation demand and supply for the period 1986–2016. Source: MEW [32].

Year	Peak Demand (MW)	Installed Capacity (MW)
1986	3480	5386
1996	5200	6898
2006	8900	10,229
2016	13,390	18,870

**Table 2:** International market prices and Kuwait’s MEW cost prices of fuels used in power stations for the year 2016. Source: IEA [30], MEW [32], and Lazard [33].

Fuel Category	International Market Price (USD)	Kuwait MEW Cost (USD)	Unit
Heavy Oil	20–38	35	Barrel (based on API)
Natural Gas	3.55	3	MMBTU
Crude Oil	51	40	Barrel *
Gas Oil	53.3	53	Barrel

* Conversion factor of OPEC (1 ton: 7.33 barrel) is used to convert gas oil price USD 390.61/MT to USD 53.3/barrel.

**Table 3:** Total cost of the selected CSMs for the power station. Source: NETL [39,40,41].

Category	MW-Net	TASC	OM	TS&M	TC
U1	543.3	USD 2,530,832	USD 231,344	USD 138,109	USD 2,900,285
U2	473.6	USD 940,497	USD 208,460	USD 57,448	USD 1,206,405
C1	471.6	USD 2,286,707	USD 126,685	USD 120,670	USD 2,534,062
C2	500.1	USD 2,495,874	USD 135,120	USD 131,550	USD 2,762,544
C3	460.9	USD 1,939,291	USD 109,389	USD 102,434	USD 2,151,114
C4	445.3	USD 2,191,925	USD 121,312	USD 115,662	USD 2,428,899
C5	466.5	USD 2,355,769	USD 127,483	USD 124,163	USD 2,607,415
C6	515.1	USD 2,261,041	USD 138,146	USD 119,959	USD 2,519,146
CB1	497.0	USD 2,784,423	USD 228,275	USD 150,635	USD 3,163,333
CB2	514.0	USD 2,567,836	USD 231,281	USD 139,956	USD 2,939,073
Average	489	USD 2,235,420	USD 165,749	USD 120,058	USD 2,521,227

**Table 4:** Total cost of the selected CSMs for the CCSF. Source: NETL [39,40,41].

Cases	CP	OM	TC	%
U1	USD 304,043	USD 68,793	USD 372,836	17%
U2	USD 311,405	USD 66,436	USD 377,841	43%
C1	USD 206,132	USD 49,562	USD 255,694	13%
C2	USD 226,868	USD 54,295	USD 281,163	13%
C3	USD 171,819	USD 41,250	USD 213,069	13%
C4	USD 255,542	USD 59,592	USD 315,134	16%
C5	USD 248,993	USD 58,455	USD 307,448	15%
C6	USD 249,337	USD 58,242	USD 307,579	16%
CB1	USD 264,007	USD 71,300	USD 335,307	14%
CB2	USD 335,462	USD 86,459	USD 421,921	19%
Average	USD 246,767	USD 57,078	USD 303,846	18%

**Table 5:** A summary for cost outputs for the PS-CSM.

Project	Cost (USD/Mill)	Production (MW)	COE (USD/MW)
Case Study Model	905,405	5806	155.95

**Table 6:** A summary of cost outputs for the CCSF-CSM.

Project	Cost (USD/Mill)	Production (MW)	COE (USD/MW)
Case Study Model	378,120	2246	168.38

**Table 7:** Cost adjustment (sensitivity analysis) scenarios for the CCSF.

No	Scenarios (+/−) Operational Cost (USD 856,256 per Annum)	IRR	NPV	ROI	Payback Period
3	−3%	11%	193,945	81%	11.15
2	−2%	10%	152,327	64%	11.15
1	−1%	9%	104,085	44%	12.13
Baseline (business as usual) scenario		7%	47,928	20%	13.11
4	+1%	5%	−17,713	−7%	15.08
5	+2%	1%	−94,738	−40%	19.04
6	+3%	−1%	−185,458	−78%	30

**Table 8:** Price adjustment scenarios A for the CCSF.

No	Sensitivity Scenarios	IRR	NPV	ROI	Payback Period
1	A-15	41%	1,164,774	487%	2.34
Base Study		7%	47,928	20%	13.11
2	A-25	−10%	−590,409	−247%	30

## Data Availability

Not applicable.

## Supplementary Materials

The following supporting information can be downloaded at: https://www.mdpi.com/article/10.3390/ijerph19116490/s1. References [4,18,19,20,39,40,41,65,66,67,68,69,70,71,72,73,74,75,76,77,78,79] are cited in the supplementary materials.

## Abbreviations

AC	average cost
BTU	British thermal unit
CCS	carbon capture and storage
CCSFCM	carbon capture and storage facility case model
CO_2_	carbon dioxide
COE	cost of electricity
CSM	case study models
FS	feasibility study
GO	gas oil
HO	heavy oil
IRR	internal rate of return
Kwh	kilowatt per hour
LAC	lowest average cost (USD)
LCOE	levelized cost of electricity (USD/MW)
Mtpa	million tons per annum
MW	megawatt
NPV	net present value (USD)
OCFIX	first-year operating costs (USD)
OCVAR	first-year variable operating costs (USD)
PBP	payback period (year)
PSCM	power station case model
PSIC	power station installed capacity (MW)
SAMLCAT	statistical average method on the low-cost accounting technique
TC	total cost (million USD)
TOC	first-year capital costs (charges) (USD)

## References

[1] Intergovernmental Panel on Climate Change (IPCC) (2018). Global Warming of 1.5 °C: An IPCC Special Report on the Impacts of Global Warming of 1.5 °C above Pre-Industrial Levels.

[2] European Commission (2014). EU Emission trading System (ETS) Low Carbon Fuel Standards. http://ec.europa.eu/clima/policies/transport/fuel/index_en.htm.

[3] United Nations Framework Convention on Climate Change (2015). Adoption of the Paris Agreement: Proposal by the President.

[4] Johansson D., Franck P., Berntsson T. (2013). CO_2_ capture in oil refineries: Assessment of the capture avoidance costs associated with different heat supply options in a future energy market. Energy Convers. Manag..

[5] Merchant E.F. (2018). With 43 Carbon-Capture Projects Lined Up Worldwide, Supporters Cheer Industry Momentum. https://www.greentechmedia.com/articles/read/carbon-capture-gains-momentum.

[6] Intergovernmental Panel on Climate Change (2005). Special Report on Carbon Dioxide Capture and Storage.

[7] International Energy Agency and Organization for Economic Cooperation and Development (2004). Prospects for CO_2_ Capture and Storage: Energy Technology Analysis.

[8] Koelbl B.S., van den Broek M., Wilting H.C., Sanders M.W., Bulavskaya T., Wood R., Faaij A.P., van Vuuren D.P. (2016). Socio-economic impacts of low-carbon power generation portfolios: Strategies with and without CCS for the Netherlands. Appl. Energy.

[9] Tertrais B. (2014). DIIS Policy Brief: Uranium from Niger: A Key Resource of Diminishing Importance for France. Danish Institute for International Studies (DIIS). https://www.ciaonet.org/attachments/25248/uploads.

[10] Al-Qayim K., Nimmo W., Pourkashanian M. (2015). Comparative techno-economic assessment of biomass and coal with CCS technologies in a pulverized combustion power plant in the United Kingdom. Int. J. Greenh. Gas Control.

[11] Fan J., Wei S., Zhang X., Yang L. (2020). A comparison of the regional investment benefits of CCS retrofitting of coal-fired power plants and renewable power generation projects in China. Int. J. Greenh. Gas Control.

[12] Xianzheng H., Yong S., Zhaofeng X., Yalia X., Zhea W., Hongyi C. (2017). Techno-economic performance of wind and coal-fired power with CCS joint planning. Energy Procedia.

[13] Kaza S., Yao L.C., Bhada-Tata P., Van Woerden F. (2018). What a Waste 2.0: A Global Snapshot of Solid Waste Management to 2050.

[14] Japan National Oil Corporation (1998). Investigation of CO_2_ Sources in Kuwait.

[15] Salman M., Oskui R. (2004). Selected Processes for Recovery of Crude Oils in Kuwait. Phase I: Preliminary Study.

[16] Al-Salem S.M., Ma X., Mujaibel M.M. Assessment of CO_2_ emission sources from the petroleum sector in Kuwait. Proceedings of the 21st International Petroleum Environmental Conference (IPEC).

[17] Al-Salem S.M. Investigating the global and specific carbon dioxide (CO_2_) emissions from the petroleum downstream industry of Kuwait. Proceedings of the 5th Technological Innovations Conference and Exposition.

[18] Al-Mutairi A., Smallbone A., Al-Salem S.M., Roskilly A.P. (2017). The first carbon atlas of the state of Kuwait. Energy.

[19] Al-Salem S. (2015). Carbon dioxide (CO_2_) emission sources in Kuwait from the downstream industry: Critical analysis with a current and futuristic view. Energy.

[20] Al-Salem S., Ramadan A., Al-Mourad M., Naseeb A., Dashti B. (2020). Measurement of CO_2_ Emissions from Power and Desalination Plants in Kuwait and Possibility of Carbon Capture and Storage.

[21] Kam E., Mirza Y., Chehadeh D., Ashkanani A., Marouf R., Al-Muhareb E. (2005). KNPC Air Emissions Inventory Development, Air Module Implementation and Risk Assessment.

[22] Chehadeh D., Kam E., Mirza Y., Al-Shumari B. (2004). Experience in Air Emission Inventory Development for Refineries of the Kuwait National Petroleum Company.

[23] International Energy Agency and Organization for Economic Cooperation and Development (2008). World Energy Outlook.

[24] International Energy Agency and Organization for Economic Cooperation and Development (2016). World Energy Outlook.

[25] (2011). Statistical Yearbook of Electrical Energy, State of Kuwait: Statistics Department and Information Center.

[26] Alotaibi S. (2011). Energy consumption in Kuwait: Prospects and future approaches. Energy Policy.

[27] Aljohani T.M., Alzahrani A.M. (2014). The Operation of the GCCIA HVDC Project and Its Potential Impacts on the Electric Power Systems of the Region. Int. J. Electron. Electr. Eng..

[28] (2010). Statistical Yearbook of Electrical Energy, State of Kuwait: Statistics Department and Information Center.

[29] Global CCS Institute The Costs of CCS and Other Low-Carbon Technologies in the United States: 2015 Update. https://www.globalccsinstitute.com/publications/costs-ccs-and-other-low-carbon-technologies-2015-update.

[30] International Energy Agency (2017). World Energy Outlook (WEO) 2017.

[31] Horngren C.T., Sundem G.L., Stratton W.O., Burgstahler D., Schatzberg J. (2008). Introduction to Management Accounting.

[32] (2017). Statistical Yearbook of Electrical Energy, State of Kuwait: Statistics Department and Information Center.

[33] (2018). Lazard. https://www.lazard.com/perspective/levelized-cost-of-energy-2017/.

[34] (2018). Penn State, Penn State College of Earth and Mineral Sciences—John A. Dutton E-Education Institute. https://www.e-education.psu.edu/eme801/node/560.

[35] National Energy Technology Laboratory (2001). Cost and Performance Baseline for Fossil Energy Plants Volume 3a: Low Rank Coal to Electricity: IGCC Cases.

[36] Global CCS Institute (2018). The CO_2_ Market. https://hub.globalccsinstitute.com/publications/accelerating-uptake-ccs-industrial-use-captured-carbon-dioxide/2-co2-market.

[37] Grand View Research (2018). Market Research Report-Industry Insights. https://www.grandviewresearch.com/industry-analysis/carbon-dioxide-market.

[38] Anderson D.R., Sweeney D.J., Williams T.A., Freeman J., Shoesmith E. (2009). Statistics for Business and Economics.

[39] National Energy Technology Laboratory (NETL) (2010). Cost and Performance for Low-Rank Pulverized Coal Oxy-Combustion Energy Plants September.

[40] National Energy Technology Laboratory (NETL) (2011). Cost and Performance Baseline for Fossil Energy Plants Volume 3 Executive Summary: Low Rank Coal and Natural Gas to Electricity.

[41] National Energy Technology Laboratory (NETL) (2015). Cost and Performance Baseline for Fossil Energy Plants Volume 1b: Bituminous Coal (IGCC) to Electricity, Revision 2b.

[42] David J., Herzog H. (2001). The Cost of Carbon Capture, Greenhouse Gas Control Technologies. Proceedings of the 5th International Conference on Greenhouse Gas Control Technologies.

[43] Behrens W., Hawranek P.M. (1991). Manual for the Preparation of Industrial Feasibility Studies.

[44] Amling R. (1989). Investments an Introduction to Analysis and Management.

[45] The Economist (2003). Number Guide the Essentials of Business Numeracy.

[46] Al-Salem S.M., Papageorgiou L.G., Lettieri P. (2014). Techno-economic assessment of thermo-chemical treatment (TCT) units in the Greater London area. Chem. Eng. J..

[47] Gitman L.J. (2009). Principles of Managerial Finance.

[48] National Energy Technology Laboratory (2011). Cost and Performance Baseline for Fossil Energy Plants Volume 3a: Low Rank Coal to Electricity: IGCC Cases.

[49] Emenike O., Michailos S., Finney K.N., Hughes K.J., Ingham D., Pourkashanian M. (2020). Initial techno-economic screening of BECCS technologies in power generation for a range of biomass feedstock. Sustain. Energy Technol. Assess..

[50] Bellotti D., Sorce A., Rivarolo M., Magistri L. (2019). Techno-economic analysis for the integration of a power to fuel system with a CCS coal power plant. J. CO_2_ Util..

[51] Hondo H. (2005). Life cycle GHG emission analysis of power generation systems: Japanese case. Energy.

[52] United Nations Environment Programme (2007). Environmental Impacts of the Arab Oil and Gas Sector.

[53] Duan H., Fan Y., Zhu L. (2013). What’s the most cost-effective policy of CO_2_ targeted reduction: An application of aggregated economic technological model with CCS?. Appl. Energy.

[54] Machado P.G., Hawkes A., de Oliveira Ribeiro C. (2021). What is the future potential of CCS in Brazil? An expert elicitation study on the role of CCS in the country. Int. J. Greenh. Gas Control.

[55] Fan J., Xua M., Wei S., Zhong P., Zhang X., Yang Y., Wang H. (2018). Evaluating the effect of a subsidy policy on carbon capture and storage (CCS) investment decision-making in China—A perspective based on the 45Q tax credit. Energy Procedia.

[56] Hong W.Y. (2022). A techno-economic review on carbon capture, utilisation and storage systems for achieving a net-zero CO_2_ emissions future. Carbon Capture Sci. Technol..

[57] Paltsev S., Morris J., Kheshgi H., Herzog H. (2021). Hard-to-Abate Sectors: The role of industrial carbon capture and storage (CCS) in emission mitigation. Appl. Energy.

[58] Loria P., Bright M.B.H. (2021). Lessons captured from 50 years of CCS projects. Electr. J..

[59] Le Quéré C., Jackson R.B., Jones M.W., Smith A.J.P., Abernethy S., Andrew R.M., De-Gol A.J., Willis D.R., Shan Y., Canadell J.G. (2020). Temporary reduction in daily global CO_2_ emissions during the COVID-19 forced confinement. Nat. Clim. Change.

[60] Friedlingstein P., Le Quéré C., Canadell P., Jackson R., Peters G. (2022). Impact of COVID-19 on CO_2_ Emissions. Global Carbon Project UNFCC. https://unfccc.int/sites/default/files/resource/1.GCP_.pdf.

[61] Huang S., Xiao X., Guo H. (2022). A novel method for carbon emission forecasting based on EKC hypothesis and nonlinear multivariate grey model: Evidence from transportation sector. Environ. Sci. Pollut. Res..

[62] Gao M., Yang H., Xiao Q., Goh M. (2022). A novel method for carbon emission forecasting based on Gompertz’s law and fractional grey model: Evidence from American industrial sector. Renew. Energy.

[63] Li X., Xiao X., Guo H. (2022). A novel grey Bass extended model considering price factors for the demand forecasting of European new energy vehicles. Neural Comput. Appl..

[64] Gao M., Yang H., Xiao Q., Goh M. (2022). COVID-19 lockdowns and air quality: Evidence from grey spatiotemporal forecasts. Socio-Economic Plan. Sci..

[65] Darwish A., Darwish M. (2008). Energy and water in Kuwait: A sustainability viewpoint, Part II. Desalination.

[66] Darwish M., Abdulrahim H., Amer A. (2008). On better utilization of gas turbines in Kuwait. Energy.

[67] Darwish M., Al-Awadhi F., Darwish A. (2008). Energy and water in Kuwait. A sustainability view point, Part I. Desalination.

[68] Darwish M., Al-Najem N. (2005). The water problem in Kuwait. Desalination.

[69] Darwish M. (2013). Towards energy conservation in Qatar. Open J. Energy Effic..

[70] Ferguson S., Stockle M., Stamateris B. Reducing CO_2_ carbon capture options applied to the refining industry. Proceedings of the ERTC Annual Meeting.

[71] IEA (2004). Prospects for CO_2_ Capture and Storage. Energy Technology Analysis.

[72] Ishida M., Jin H. (1994). A novel combustor based on chemical-looping combustion reactions and its reactions kinetics. J. Chem. Eng. Jpn..

[73] Ishida M., Zheng D., Akehata T. (1987). Evaluation of a chemical-looping-combustion power-generation system by graphic exergy analysis. Energy.

[74] Johansson D., Sjöblom J., Bernstsson T. (2012). Heat supply alternatives for CO_2_ capture in the process industry. Int. J. Greenh. Gas Control.

[75] Johansson D., Rootzen J., Bernstsson T., Johnsson F. (2012). Assessment of strategies for CO_2_ abetment in the European petroleum refining industry. Energy.

[76] Johansson D., Franck P., Pettersson K., Berntsson T. (2013). Comparative study of Fischer–Tropsch production and post-combustion CO_2_ capture at an oil refinery: Economic evaluation and greenhouse gas emissions (GHG) balances. Energy.

[77] Markström P., Linderholm C., Lyngfelt A. (2013). Chemical-looping combustion of solid fuels—Design and operation of a 100 kW unit with bituminous coal. Int. J. Greenh. Gas Control.

[78] MEW (2014). Statistical Yearbook of Electricity Energy.

[79] Thernesz A., Szalmas G., Dinka P., Simon T. (2008). CO_2_ Capture-new challenge in refinery industry. MOL Sci. Mag..

