# Supplementary material for: Economic Feasibility Study of a Carbon Capture and Storage (CCS) Integration Project in an Oil-Driven Economy: The Case of the State of Kuwait
Source: Int J Environ Res Public Health. 2022 May 26;19(11):6490. doi: 10.3390/ijerph19116490 (PMC9180847; doi:10.3390/ijerph19116490)
Supplement: Supplementary file 1 [file ijerph-19-06490-s001.zip › ijerph-1715668-supplementary.pdf]

Supplementary Material File:

This file contains the associated data and complementary material as referred to in the article.

Table S1. Profile of Worldwide Large Power Sector Carbon Dioxide (CO_2) stationary sources emitting more than 0.1 Mt CO_2 yr^{-1}*. Adapted from IPCC (2005) and Al-Salem (2015).

Process	CO_2 concentration in gas stream (vol.%)	Number of sources	Emission (Million tpa)	% of total emission	Average emission per source ($MtCO_2$ per source)
Power Sector					
Coal	12 to 15	2,025	7,984	59.69	3.94
Natural gas	7-10	1,728	1,511	11.3	0.77-1.01
Fuel oil	3-8	1,108	980	10.51	0.55-1.27
Other fuels+	NA	79	61	0.45	0.77
Hydrogen	NA	2	3	0.02	1.27

*The definition of large sources is set as a carbon emitter of over 0.1 Mt CO_2 yr^{-1} (IPCC, 2005); +Other fuels include: Other gases or oil, digester gas and landfill gas.

Table S2. Profile of Carbon Sources Excluding Power Sector. Adapted from IPCC (2005) and Al-Salem (2015).

Process	CO_2 concentration in gas stream (vol.%)	Number of sources	Emission (Million tpa)	% of total emission	Average emission per source ($MtCO_2$ per source)
Natural gas sweetening	NA++	NA	50+++	0.37	NA
Petrochemical industry					
Ethylene	12	240	258	1.93	1.08
Ammonia: Process	100	194	113	0.84	0.58
Ammonia: Fuel Combustion	8	19	5	0.04	0.26
Ethylene Oxide (EO)	100	17	3	0.02	0.15
Total of petrochemical sector	220	470	379	2.83	-
Cement production	20	1,175	932	6.97	0.79
Petroleum refineries	3 to 13	638	798	5.97	1.25
Biomass (bioenergy and fermentation)	3 to 100	303	13,466	-	0.2

Table S3. Properties of candidate gas streams that can be used as input to a capture process. Adapted from: IEA (2004).

Source	CO_2 concentration (% dry by volume)	Pressure of gas stream (MPa)	CO_2 partial pressure (MPa)
CO_2 from fuel combustion			
Power station flue gas			
Natural gas fired boilers	7 - 10	0.1	0.007 - 0.010
Gas turbines	3 - 4		0.003 - 0.004
Oil fired boilers	11 - 13		0.011 - 0.013
Coal fired boilers	12 - 14		0.012 - 0.014

Table S4. Summary of main carbon capture techniques (CCT). After Al-Salem (2015); Al-Mutairi et al. (2017), Thernesz et al. (2008); Johansson et al. (2012; 2013), Fergusson et al. (2011).

Technique	Process Summary	Unit Operation/Technologies/Examples
Post-combustion capture	• Noted to be the most mature technique which utilizes capture of CO_2 emitted from combustion of fossil fuels in air.<br>• The flue gas of the combustion process is used as the feed to this technique.<br>• This technique uses technologies well defined in process industry for gas separation where flue gas is typically washed with physical solvent. The gas is scrubbed up to 90% of its CO_2 content.	• The use of solvents and sorbents is considered as prime example for extracting carbon rich streams after passing CO_2 rich gas in intimate contact with solvents or sorbents capable of capturing it.<br>• Application of polymeric, metallic or ceramic membrane is another common method.<br>• A range of solvents exists namely monoethanolamine (MEA), ammonia or seawater.
Pre-combustion capture	• The technology relies on decarbonizing the fuel gas before entering the combustion process with a H_2 rich one.<br>• Otherwise, a central location might be used to collect and reform the feed gas, where all inlet gas is decarbonized. This method is best used when refinery gas is used for combined heat and power (CHP) generation or for power stations using refinery feedstock (Fergusson et al., 2011). Solvent removal can be achieved by Selexol, methyl-diethanolamine (MDEA) or membranes.	• The reaction of fuel and oxygen post syngas production ($CO + H_2$) is achieved. Absorption by physical or chemical means is then applied.<br>• The CO is then reacted with steam (catalytically) in a shift converter to produce CO_2 (later separated) and H_2O. A range of coals, petcoke, fuel oils or solid waste can be used as gasifier feedstock.
Oxy fuel combustion	• This technique utilises the concept of combusting fuel (with oxygen) from an air separation unit.<br>• Boiler temperature is typically moderated by using a recycled portion of the flue gas back to the combustion chamber in such technologies.<br>• The produced CO_2 and/or H_2O rich gas can be recycled to the combustor to reduce the concentration of oxygen.	This method is suitable for units, where pure O_2 is used instead of air in combustion processes to produce CO_2 and water instead of a mixture of CO_2, N_2 and water.
Chemical Looping	• Noted to be the least mature technique in CCS with majority of the work in research and pilot stages.<br>• The concept relies on the circulation of an oxygen carrier particle (e.g. metal oxide) between an air reactor and a fuel reactor (Markström et al., 2013), where particles are then transported through a loop seal, entering the fuel reactor where CO_2 is fluidized.	• The name was originally recognised as chemical looping back in 1987 (Ishida and Jin, 1994; Ishida et al., 1987).<br>• Typically, active oxides of iron, nickel, copper and manganese are used as carriers. Gaseous fuels are typically used and success was reported for more than 4000 h of operation in 12 units of 0.3-140 kW.

Table S5. Power stations in Kuwait with respect to fuel used and type of turbine. Source: MEW (2014).

Power Station	Declared Capacity (MW output)	Primary Fuel Used	Type of Turbine
Shuwaikh	252	Natural gas	Gas cycle
Shuaiba North	876	Natural gas and Gas oil	Gas cycle
Shuaiba South	720	Natural gas	Thermal steam turbine
Doha East	1158	Natural gas and Gas oil/Gas oil and Crude oil	Gas cycle and Thermal steam turbine
Doha West	2360	Natural gas and Heavy oil	
Al-Zour	5306	Natural gas and Gas oil/Gas oil and Crude oil	
Sabiya	4867		

Table S6. Power stations in Kuwait with respect to type of turbine and capacity distribution. Source: MEW (2014).

Stations[a]	Current available capacity				Total available capacity
	Gas turbine[d]		Steam turbine		
	Capacity of each unit	Total[e]	Capacity of each unit	Total	
Shuwaikh station[b]	6 x 42	252	-	-	252
Shuaiba South station	-	-	6 x 120	720	720
Shuaiba North station	3 x 2 20	660	1 x 215.5	215.5	875.5
Doha East station	6 x 18	108	7 x 150	1050	1158
Doha West station	5 x 28.2	141	8 x 300	2400	2541
Al-Zour South station	8 x 130<br>4 x 27.7<br>5 x 165	1040<br>110.8<br>825	8 x 300<br>2 x 280<br>2 x 185	2400<br>560<br>370	5305.8
Sabiya station[c]	6 x 41.7<br>4 x 62.5<br>6 x 220	250.2<br>250<br>1320	8 x 300<br>3 x 215.5	2400<br>646.5	4866.7
Total		4957		10762	15719

Note to reader: a. Thermal steam turbines are dominant type due to their high efficiency steam cycle. Combine cycle turbines are also hosted including both steam and gas turbines to obtain the desired electrical output. The constructed complementary gas turbines are aimed at overcoming peak loads at summer season; b. One reverse osmosis (RO) unit exists in this station and has been included in the analysis in combination with the electrical generation capacity to reflect the real-life scenario of the plant. All stations include desalination units which represent 93% of fresh water supply (Darwish et al., 2008a; 2008b; Darwish and Al-Najem, 2005; Darwish and Darwish, 2008). c. Sabyia power station operates a steam cycle with an efficiency ranging between 30 to 35% (Al-Salem et al., 2020); d. It can be noted that the main contributor to the emission strength in Kuwait is Al-Zour station (32%) (Figure 1 in Article). As shown the station utilises the four main types of fossil fuels used for the power sector in Kuwait (e.g., natural gas, heavy fuel oil, gas oil and crude oil). The largest consumption in the station is dedicated for heavy fuel oil to feed the gas turbines by the amount of 1.009×10^6 tpa. This is closely followed by crude oil consumption for the turbines of the station by the amount of 824.363 x 103 tpa. The second largest emitter among all stations is Sabyia station, which also consumes heavy fuel oil as the main fossil fuel for its turbines by the amount of 1.89×10^6 mtpa. Each of those stations host eight steam turbines of a 300 MW capacity that uses heavy fuel oil as the main fuel component (Al-Mutairi et al., 2017; Al-Salem et al., 2020); e. Natural gas for the power sector is consumed at the amount of 20.1 bcm and deficit is imported from neighbouring countries (WEC, 2017). Petroleum refineries and their hydrocarbon flaring activities are depicted elsewhere (Al-Salem, 2015).

Table S7. Fuel consumed in Kuwait's power sector during the years 2010-2013 and emission factors (EF) for each type of fuel used.

Fuel (million ton) (MEW, 2014; Al-Mutairi et al., 2017)	2010	2011	2012	2013	EF (kg CO_2/kg fuel) (Darwish, 2013)
Gas oil	1.0241	1.154	1.342	1.0410	0.875
Heavy fuel oil	4.947	4.387	4.345	5.293	0.85
Natural gas	5.285	6.080	6.654	6.387	0.75
Crude oil	2.0244	2.191	1.867	1.276	0.85

Table S8. Power Station Raw Materials.

Category	Value ($)
COAL & SORBENT HANDLING	3,249
COAL & SORBENT PREP & FEED	5,517
FEED WATER & MISC. BOP SYSTEMS	7,230
GASIFIER & ACCESSORIES	12,187
GAS CLEANUP & PIPING & CO2 COMPRESSION	3,037
COMBUSTION TURBINE/ACCESSORIES	806
HRSG, DUCTING & STACK	2,436
STEAM TURBINE GENERATOR	1,059
COOLING WATER SYSTEM	8,140
ASH/SPENT SORBENT HANDLING SYS	1,623
ACCESSORY ELECTRIC PLANT	13,137
INSTRUMENTATION & CONTROL	2,069
IMPROVEMENTS TO SITE	2,045
BUILDINGS & STRUCTURES	6,957
Initial Cost for Catalyst and Chemicals	7,532
Inventory Capital	16,084
Total	93,108

Table S9. Power Station Equipment's.

Category	Value ($)
COAL & SORBENT HANDLING	17,484
COAL & SORBENT PREP & FEED	30,185
FEED WATER & MISC. BOP SYSTEMS	8,916
GASIFIER & ACCESSORIES	377,533
GAS CLEANUP & PIPING & CO2 COMPRESSION	111,559
COMBUSTION TURBINE/ACCESSORIES	92,027
HRSG, DUCTING & STACK	34,963
STEAM TURBINE GENERATOR	70,034
COOLING WATER SYSTEM	8,342
ASH/SPENT SORBENT HANDLING SYS	21,401
ACCESSORY ELECTRIC PLANT	33,841
INSTRUMENTATION & CONTROL	11,248
IMPROVEMENTS TO SITE	3,470
Total Cost	821,003

Table S10. Power Station Labour.

Category	Value ($)
Labour	323,505

Table S11. Power Station Utilities

Utilities	Value ($)
Water consumption	3,240
Electricity Consumption	350
Total	3,590

Table S12. Power Station Structures & Buildings

Building Type	Value ($)
Combustion Turbine Area	541
Steam Turbine Building	7,796
Administration Building	1,995
Circulation Water Pumphouse	316
Water Treatment Buildings	1,135
Machine Shop	949
Warehouse	1,497
Other Buildings & Structures	1,012
Waste Treating Building & Str.	3,716
Total	18,958

Table S13. Power Station Fixed Assets

Fixed Capital	Value ($)
Construction Cost	
Building & Structures	18,958
Equipment's	821,003
Total	839,961

Table S14. Power Station Depreciation

Depreciation	Original Value	Service Life	1	2	3	4	5	6	7	8	9	10
Building	18,958	30	632	632	632	632	632	632	632	632	632	632
Equipment's	821,003	30	27,367	27,367	27,367	27,367	27,367	27,367	27,367	27,367	27,367	27,367
Total Depreciation	839,961		27,999	27,999	27,999	27,999	27,999	27,999	27,999	27,999	27,999	27,999
Depreciation	Original Value	Service Life	11	12	13	14	15	16	17	18	19	20
Building	18,958	30	632	632	632	632	632	632	632	632	632	632
Equipment's	821,003	30	27,367	27,367	27,367	27,367	27,367	27,367	27,367	27,367	27,367	27,367
Total Depreciation	839,961		27,999	27,999	27,999	27,999	27,999	27,999	27,999	27,999	27,999	27,999
Depreciation	Original Value	Service Life	21	22	23	24	25	26	27	28	29	30
Building	18,958	30	632	632	632	632	632	632	632	632	632	632
Equipment's	821,003	30	27,367	27,367	27,367	27,367	27,367	27,367	27,367	27,367	27,367	27,367
Total Depreciation	839,961		27,999	27,999	27,999	27,999	27,999	27,999	27,999	27,999	27,999	27,999

Table S15. Power Station Total Cost

Category / Years	1	2	3	4	5	6	7	8	9	10
Operating Costs	856,256	856,256	856,256	856,256	856,256	856,256	856,256	856,256	856,256	856,256
Factory Costs										
O&M	102,152	102,152	102,152	102,152	102,152	102,152	102,152	102,152	102,152	102,152
TS & M	148,593	148,593	148,593	148,593	148,593	148,593	148,593	148,593	148,593	148,593
Fuel	593,589	593,589	593,589	593,589	593,589	593,589	593,589	593,589	593,589	593,589
Total Factory Cost (Operational Cost)	844,335	844,335	844,335	844,335	844,335	844,335	844,335	844,335	844,335	844,335
Non-factory Cost										
Eng'g CM H.O.& Fee	3,794	3,794	3,794	3,794	3,794	3,794	3,794	3,794	3,794	3,794
Land	30	30	30	30	30	30	30	30	30	30
Other Owner's Costs	8,098	8,098	8,098	8,098	8,098	8,098	8,098	8,098	8,098	8,098
Total Non-Factory Cost (Fixed Cost)	11,922	11,922	11,922	11,922	11,922	11,922	11,922	11,922	11,922	11,922
Non-Operating Costs (Capital Cost)										
Financing Costs	1,458	1,458	1,458	1,458	1,458	1,458	1,458	1,458	1,458	1,458
Interest Value Industrial	19,693	17,723	17,594	17,464	17,334	17,204	17,074	16,945	16,815	16,685
Interest Commercial		22,975	20,103	19,914	19,724	19,535	19,346	19,156	18,967	18,778
Depreciation and Amortization	27,999	27,999	27,999	27,999	27,999	27,999	27,999	27,999	27,999	27,999
Total Non-Operation Costs	49,149	70,154	67,153	66,834	66,515	66,195	65,876	65,557	65,238	64,919
Total Production Costs	905,405	926,410	923,409	923,090	922,771	922,452	922,133	921,814	921,495	921,175

Table S15. (Cont'd) Power Station Total Cost

Category / Years	11	12	13	14	15	16	17	18	19	20
Operating Costs	856,256	856,256	856,256	856,256	856,256	856,256	856,256	856,256	856,256	856,256
Factory Costs										
O&M	102,152	102,152	102,152	102,152	102,152	102,152	102,152	102,152	102,152	102,152
TS & M	148,593	148,593	148,593	148,593	148,593	148,593	148,593	148,593	148,593	148,593
Fuel	593,589	593,589	593,589	593,589	593,589	593,589	593,589	593,589	593,589	593,589
Total Factory Cost (Operational Cost)	844,335	844,335	844,335	844,335	844,335	844,335	844,335	844,335	844,335	844,335
Non-factory Cost										
Eng'g CM H.O.& Fee	3,794	3,794	3,794	3,794	3,794	3,794	3,794	3,794	3,794	3,794
Land	30	30	30	30	30	30	30	30	30	30
Other Owner's Costs	8,098	8,098	8,098	8,098	8,098	8,098	8,098	8,098	8,098	8,098
Total Non-Factory Cost (Fixed Cost)	11,922	11,922	11,922	11,922	11,922	11,922	11,922	11,922	11,922	11,922
Non-Operating Costs (Capital Cost)										
Financing Costs	1,458	1,458	1,458	1,458	1,458	1,458	1,458	1,458	1,458	1,458
Interest Value Industrial	-	-	-	-	-	-	-	-	-	-
Interest Commercial	18,589	-	-	-	-	-	-	-	-	-
Depreciation and Amortization	27,999	27,999	27,999	27,999	27,999	27,999	27,999	27,999	27,999	27,999
Total Non-Operation Costs	48,045	29,456	29,456	29,456	29,456	29,456	29,456	29,456	29,456	29,456
Total Production Costs	904,301	885,712	885,712	885,712	885,712	885,712	885,712	885,712	885,712	885,712

Table S15. (Cont'd) Power Station Total Cost

Category / Years	21	22	23	24	25	26	27	28	29	30
Operating Costs	856,256	856,256	856,256	856,256	856,256	856,256	856,256	856,256	856,256	856,256
Factory Costs										
O&M	102,152	102,152	102,152	102,152	102,152	102,152	102,152	102,152	102,152	102,152
TS & M	148,593	148,593	148,593	148,593	148,593	148,593	148,593	148,593	148,593	148,593
Fuel	593,589	593,589	593,589	593,589	593,589	593,589	593,589	593,589	593,589	593,589
Total Factory Cost (Operational Cost)	844,335	844,335	844,335	844,335	844,335	844,335	844,335	844,335	844,335	844,335
Non-factory Cost										
Eng'g CM H.O.& Fee	3,794	3,794	3,794	3,794	3,794	3,794	3,794	3,794	3,794	3,794
Land	30	30	30	30	30	30	30	30	30	30
Other Owner's Costs	8,098	8,098	8,098	8,098	8,098	8,098	8,098	8,098	8,098	8,098
Total Non-Factory Cost (Fixed Cost)	11,922	11,922	11,922	11,922	11,922	11,922	11,922	11,922	11,922	11,922
Non-Operating Costs (Capital Cost)										
Financing Costs	1,458	1,458	1,458	1,458	1,458	1,458	1,458	1,458	1,458	1,458
Interest Value Industrial	-	-	-	-	-	-	-	-	-	-
Interest Commercial	-	-	-	-	-	-	-	-	-	-
Depreciation and Amortization	27,999	27,999	27,999	27,999	27,999	27,999	27,999	27,999	27,999	27,999
Total Non-Operation Costs	29,456	29,456	29,456	29,456	29,456	29,456	29,456	29,456	29,456	29,456
Total Production Costs	885,712	885,712	885,712	885,712	885,712	885,712	885,712	885,712	885,712	885,712

Table S16. PS Pre-Production

Project Management	Value ($)
6 Months Fixed O&M	15,260
1 Month Variable O&M	4,088
25% of 1 Months Fuel Cost at 100% CF	937
2% of TPC	32,390
Total	52,676

Table S17. PS Working Capital

Category	Value ($)
Salaries and Wages	323,505
Raw Material	93,108
Utilities	3,590
Total	420,203

Table S18. PS Initial Investment

Category	Value ($)
Total Fixed Assets	839,961
Working Capital	420,203
Pre-production Expenditure	52,676
Total	1,312,840

Table S19. Average HO Prices for MEW-PS.

PS	Prices HO ($)
Doha West	34.864
Al-Zour South	33.834
Average	34.349

Table S20. Power Station Revenue

Category / Years	1	2	3	4	5	6	7	8	9	10
Production /ton	5,806	5,806	5,806	5,806	5,806	5,806	5,806	5,806	5,806	5,806
LCOE	175.000	175.000	175.000	175.000	175.000	175.000	175.000	175.000	175.000	175.000
Total Revenue	1,016,015	1,016,015	1,016,015	1,016,015	1,016,015	1,016,015	1,016,015	1,016,015	1,016,015	1,016,015
Category / Years	11	12	13	14	15	16	17	18	19	20
Production /ton	5,806	5,806	5,806	5,806	5,806	5,806	5,806	5,806	5,806	5,806
LCOE	175.000	175.000	175.000	175.000	175.000	175.000	175.000	175.000	175.000	175.000
Total Revenue	1,016,015	1,016,015	1,016,015	1,016,015	1,016,015	1,016,015	1,016,015	1,016,015	1,016,015	1,016,015
Category / Years	21	22	23	24	25	26	27	28	29	30
Production /ton	5,806	5,806	5,806	5,806	5,806	5,806	5,806	5,806	5,806	5,806
LCOE	175.000	175.000	175.000	175.000	175.000	175.000	175.000	175.000	175.000	175.000
Total Revenue	1,016,015	1,016,015	1,016,015	1,016,015	1,016,015	1,016,015	1,016,015	1,016,015	1,016,015	1,016,015

Table S21. Power Station Cash Flow.

Category	Construction Period	1	2	3	4	5	6	7	8	9	10
Cash In	1,312,840	1,044,014	1,044,014	1,044,014	1,044,014	1,044,014	1,044,014	1,044,014	1,044,014	1,044,014	1,044,014
Sales Revenue	-	1,016,015	1,016,015	1,016,015	1,016,015	1,016,015	1,016,015	1,016,015	1,016,015	1,016,015	1,016,015
Investor	328,210	-	-	-	-	-	-	-	-	-	-
Commercial Bank Loan	328,210	-	-	-	-	-	-	-	-	-	-
Industrial Bank Loan	656,420	-	-	-	-	-	-	-	-	-	-
Depreciation & Amortization	-	27,999	27,999	27,999	27,999	27,999	27,999	27,999	27,999	27,999	27,999
Cash Out	1,312,840	908,770	981,170	901,524	901,204	900,885	900,566	900,247	899,928	899,609	899,290
Initial Investment	1,312,840	-	-	-	-	-	-	-	-	-	-
Payment of Replacement	-	-	-	-	-	-	-	-	-	-	-
Operating Cost	-	856,256	856,256	856,256	856,256	856,256	856,256	856,256	856,256	856,256	856,256
Principal Repayment Commercial Bank	-	32,821	2,163	2,163	2,163	2,163	2,163	2,163	2,163	2,163	2,163
Principal Repayment Industrial Bank	-		82,053	5,408	5,408	5,408	5,408	5,408	5,408	5,408	5,408
Interest Repayment Commercial Bank	-	19,693	17,723	17,594	17,464	17,334	17,204	17,074	16,945	16,815	16,685
Interest Repayment Industrial Bank	-		22,975	20,103	19,914	19,724	19,535	19,346	19,156	18,967	18,778
Net Cash Flow	-1,312,840	135,244	62,844	142,490	142,809	143,128	143,447	143,766	144,085	144,405	144,724

Category	Construction Period	11	12	13	14	15	16	17	18	19	20
Cash In	1,312,840	1,044,014	1,044,014	1,044,014	1,044,014	1,044,014	1,044,014	1,044,014	1,044,014	1,044,014	1,044,014
Sales Revenue	-	1,016,015	1,016,015	1,016,015	1,016,015	1,016,015	1,016,015	1,016,015	1,016,015	1,016,015	1,016,015
Investor	328,210	-	-	-	-	-	-	-	-	-	-
Commercial Bank Loan	328,210	-	-	-	-	-	-	-	-	-	-
Industrial Bank Loan	656,420	-	-	-	-	-	-	-	-	-	-
Depreciation & Amortization	-	27,999	27,999	27,999	27,999	27,999	27,999	27,999	27,999	27,999	27,999
Cash Out	1,312,840	880,253	856,256	856,256	856,256	856,256	856,256	856,256	856,256	856,256	856,256
Initial Investment	1,312,840	-	-	-	-	-	-	-	-	-	-
Payment of Replacement	-	-	-	-	-	-	-	-	-	-	-
Operating Cost	-	856,256	856,256	856,256	856,256	856,256	856,256	856,256	856,256	856,256	856,256
Principal Repayment Commercial Bank	-	-	-	-	-	-	-	-	-	-	-
Principal Repayment Industrial Bank	-	5,408	-	-	-	-	-	-	-	-	-
Interest Repayment Commercial Bank	-	-	-	-	-	-	-	-	-	-	-
Interest Repayment Industrial Bank	-	18,589	-	-	-	-	-	-	-	-	-
Net Cash Flow	-1,312,840	163,761	187,758	187,758	187,758	187,758	187,758	187,758	187,758	187,758	187,758

Category	Construction Period	21	22	23	24	25	26	27	28	29	30
Cash In	1,312,840	1,044,014	1,044,014	1,044,014	1,044,014	1,044,014	1,044,014	1,044,014	1,044,014	1,044,014	1,044,014
Sales Revenue	-	1,016,015	1,016,015	1,016,015	1,016,015	1,016,015	1,016,015	1,016,015	1,016,015	1,016,015	1,016,015
Investor	328,210	-	-	-	-	-	-	-	-	-	-
Commercial Bank Loan	328,210	-	-	-	-	-	-	-	-	-	-
Industrial Bank Loan	656,420	-	-	-	-	-	-	-	-	-	-
Depreciation & Amortization	-	27,999	27,999	27,999	27,999	27,999	27,999	27,999	27,999	27,999	27,999
Cash Out	1,312,840	856,256	856,256	856,256	856,256	856,256	856,256	856,256	856,256	856,256	856,256
Initial Investment	1,312,840	-	-	-	-	-	-	-	-	-	-
Payment of Replacement	-	-	-	-	-	-	-	-	-	-	-
Operating Cost	-	856,256	856,256	856,256	856,256	856,256	856,256	856,256	856,256	856,256	856,256
Principal Repayment Commercial Bank	-	-	-	-	-	-	-	-	-	-	-
Principal Repayment Industrial Bank	-	-	-	-	-	-	-	-	-	-	-
Interest Repayment Commercial Bank	-	-	-	-	-	-	-	-	-	-	-
Interest Repayment Industrial Bank	-	-	-	-	-	-	-	-	-	-	-
Net Cash Flow	-1,312,840	187,758	187,758	187,758	187,758	187,758	187,758	187,758	187,758	187,758	187,758

References

Al-Mutairi, A., Smallbone, A., Al-Salem, S.M., Roskilly, A.P., 2017. The First Carbon Atlas of the State of Kuwait, Energy, 133: 317-326.

Al-Salem, S., A. Ramadan, M. Al-Mourad, A. Naseeb, B. Dashti, 2020. Measurement of CO2 Emissions from Power and Desalination Plants in Kuwait and Possibility of Carbon Capture and Storage. KISR Final Report, EC093C, Kuwait.

Al-Salem, S.M., 2015. Carbon dioxide (CO_2) emission sources in Kuwait from the downstream industry: Critical analysis with a current and futuristic view. Energy, 81: 575-587.

Darwish A, Darwish M., 2008. Energy and water in Kuwait: A sustainability viewpoint Part II, Desalination, 230: 140-152.

Darwish M, Abdulrahim H, Amer A., 2008a. On better utilization of gas turbines in Kuwait. Energy 55: 571-588.

Darwish M, Al-Awadhi F, Darwish A., 2008b. Energy and water in Kuwait. A sustainability view point Part I. Desalination, 225; 341-355.

Darwish M, Al-Najem N., 2005. The water problem in Kuwait. Desalination, 177: 167-177.

Darwish M., 2013. Towards energy conservation in Qatar. Open Journal of Energy Efficiency 2: 176-191.

Ferguson, S., M. Stockle, B. Stamateris, 2011. Reducing CO2 carbon capture options applied to the refining industry. Foster Wheeler technologies, Proceedings of ERTC Annual Meeting, Barcelona, 29 November-1 December.

IEA, 2004. Prospects for CO_2 Capture and Storage. Energy Technology Analysis. International Energy Agency and Organization for Economic Cooperation and Development.

IPCC, 2005. Intergovernmental Panel on Climate Change Special Report on Carbon Dioxide Capture and Storage. IPCC Special Report on CO_2 Capture and Storage.

Ishida, M., Jin, H., 1994. A novel combustor based on chemical-looping combustion reactions and its reactions kinetics. Journal of Chemical Engineering of Japan 27(3): 296–301.

Ishida, M., Zheng, D., Akehata, T., 1987. Evaluation of a chemical-looping-combustion power-generation system by graphic exergy analysis. Energy 12(2): 147–154.

Johansson, D., J. Sjöblom, T. Bernstsson, 2012. Heat supply alternatives for CO2 capture in the process industry. International Journal of Greenhouse Gas Control 8: 217–232.

Johansson, D., P. Franck, T. Bernstsson, 2013. CO2 capture in oil refineries: assessment of the capture avoidance costs associated with different heat supply options in a future energy market. Energy Conversion and Management, 66: 127–142.

Johansson, D.; J. Rootzen; T. Bernstsson; F. Johnsson, 2012. Assessment of strategies for CO2 abetment in the European petroleum refining industry. Energy 42: 375–386.

Johansson, D.; P. Franck; K. Pettersson; T. Berntsson, 2013. Comparative study of Fischer–Tropsch production and post-combustion CO2 capture at an oil refinery: economic evaluation and greenhouse gas emissions (GHG) balances. Energy 59: 387-401.

Markström, P., C. Linderholm, A. Lyngfelt, 2013. Chemical-looping combustion of solid fuels - Design and operation of a 100 kW unit with bituminous coal. International Journal of Greenhouse Gas Control 15: 150–162.

MEW, 2014. Statistical Yearbook of Electricity Energy. Statistics Department and Information Center, Ministry of Electricity and Water, MEW, State of Kuwait.

NETL, 2010. National Energy Technology Laboratory (NETL), Cost and Performance for Low-Rank Pulverized Coal Oxy-combustion Energy Plants September, US Department of Energy.

NETL, 2011. National Energy Technology Laboratory (NETL), Cost and Performance Baseline for Fossil Energy Plants Volume 3 Executive Summary: Low Rank Coal and Natural Gas to Electricity, US Department of Energy.

NETL, 2015. National Energy Technology Laboratory (NETL), Cost and Performance Baseline for Fossil Energy Plants Volume 1b: Bituminous Coal (IGCC) to Electricity, U.S Department of Energy, Revision 2b, July 2015.

Thernesz, A.; G. Szalmas; P. Dinka; and T. Simon. 2008. CO2 Capture-new challenge in refinery industry. MOL Scientific Magazine (Challenge), 3: 12–24.

World Energy Council (WEC), 2017. Energy resources data (Kuwait), https://www.worldenergy.org/data/resources/country/kuwait/gas/.

it is critical to clearly define the sources that will be included from the onset. For Power and Desalination Plants, the typical thermal units are the main CO_2 emission sources, in addition to the gas turbine units (GTU) that were installed over the last decade to boost the MEW production capacity. MEW P&D plants have long-term logs of the fuel types, their chemical compositions including the calorific values, and their consumption rates. This information will be obtained from the plant operators to calculate CO_2 emissions. The current available power generation capacity from the facilities which below to MEW are listed previously in Table S6. The analysis shown in this section compliments the work shown in the previous one. The latest version of the IPCC inventory software (i.e., V2.54) was utilized. In essence IPCC Inventory Software uses the following generic equation to calculate the emissions:

$$Emissions = Activity\ Rate\ \times Emisson\ Factor \qquad (1)$$

The software is built around a basic data model that relies on predetermined emission factors for different activities which have been categorized depending on the industries, e.g. energy, waste, agriculture and forestry, etc, they belong to. The software supports other database-related features like data reporting and data import/export.

In addition to using IPCC to calculate the CO_2 emissions, the latest US-EPA AP-42 emission factors were used. The AP-42 emission factors have been developed from the source test data, engineering approximations and material balance studies. The AP-42 contains process information and emission factors of more 200 emission sources, and accordingly it covers all the sources of MEW. Finally, in addition to the above two methods, the stoichiometric equations along with the fuel composition were used as a third method to calculate the CO_2 emissions form the MEW power generation facilities. The estimation of the carbon load was conducted in a systematic approach thus: Step 1) For the three methods, the process started by calculating the required thermal energy for each unit of those listed in Table S6, assuming each unit was operating at full production capacity over the whole year. Step 2) Then based on the fuel types that can be used by each unit and assuming entire reliance on this fuel; the amount of fuel that needs to be consumed by the unit was calculated. The calculations in this step relied on the net calorific value for each fuel type. Step 3A) Then based on the fuel consumption rates, the fuel chemical composition and stoichiometric equations, the amount of CO_2 emissions were calculated. Step 3B) Using the suitable US-EPA emission factor, the amount of CO_2 emissions was calculated. In addition to the U-EPA emission factor, more emission factors were used in this step and this included: 1) Ecometrica (Kuwait specific), 2) US-EPA Electricity Emission Factor (US AVG), and 3) IEA-Electricity Emission Factors 1999-2002 (Kuwait specific). Accordingly, this step led to four CO_2 emission values. Step 3C) The third calculation method was that of the IPCC. Step 4) Then based on the practical fuel combinations, e.g. NG for GTUs and HFO for the thermal units compared to GO for GTUs and HFO for the thermal units), the CO_2 emissions obtained in steps 3A, 3B and 3C were added leading to the total CO_2 emissions form each power station. Based on the above, six CO_2 emission values were obtained for each scenario. These values represent: Stoichiometric equations; US-

EPA emissions factors, Ecometrica emissions factors (Kuwait specific), US-averaged electricity emission factors, IEA emissions factors (Kuwait specific), and; IPCC emissions factors. Figure S1 shows the results obtained for Subyia Power Station. Four scenarios were considered for this station:

- the GTUs rely entirely on gas oil (GO) while the thermal units rely entirely on heavy fuel oil (HFO),
- the GTUs rely entirely on natural gas (NG) while the thermal units rely entirely on HFO,
- the GTUs rely entirely on GO while the thermal units rely entirely on fuel oil (FO), and;
- the GTUs rely entirely on NG while the thermal units rely entirely on FO.

The calculated annual CO_2 emissions ranged between 24.4 x 10^6 tons for scenario 2 to 28.6 x 10^6 tons for scenario 3 for the stoichiometric equations calculations. As for the results obtained using the US-EPA emission factors, the annual CO_2 emissions ranged between 21.9 x 10^6 tons for scenario 2 to 27.9 x 10^6 tons for scenario 3. For the Ecometrica (Kuwait specific), US-Average electricity emission factors, and the IEA Electricity emission factors (Kuwait specific), the annual CO_2 emissions were 27.2 x 10^6, 23.8 x 10^6 and 33.7 x 10^6 tons, respectively, with no difference in the results of different scenarios. Finally, the IPCC calculations resulted in annual CO_2 emissions ranging between 23.8 x 10^6 tons for Scenario 2 to 28.9 x 10^6 tons for scenario 3 (Figure S1). Using the IPCC results as a baseline, Figure S2 shows the stoichiometric equations results had the smallest percentage error (i.e., average error = 0.73%) while those obtained using the US-EPA emission factors, the Ecometrica (Kuwait specific), the US-Average electricity emission factors, and the IEA Electricity emission factors (Kuwait specific) had average percentage errors of 5.33, 4.12, 8.68, and 29.06%, respectively. Figure S3 shows the IPCC's results obtained for the above-listed four scenarios is addition to a fifth scenario reflecting the actual fuel consumed in 2016 as obtained by MEW. Obviously, a quick comparison between the emission values obtained for the five scenarios confirms the fact the units were not fully utilized throughout the year. The reader's attention is brought to the fact the vertical axis in Figure S3 is logarithmic in style, a choice made to allow the relatively negligible CH_4 and N_2O annual emissions to become visible on the chart. Figure S4 shows the results obtained for Shuaiba North Power Station (SNPS) and Shuaiba South Power Station (SSPS). Two scenarios were considered for each station:

1) the GTUs rely entirely on GO, and
2) the GTUs rely entirely on NG.

The results obtained using the stoichiometric equations calculations, the US-EPA emission factors method, and the IPCC method illustrate the fact that the CO_2 emissions resulting from the GO were higher than those obtained when burning NG. On the other hand, the results obtained from the six methods show the CO_2 emissions from SNPS were higher than those of SSPS. For the stoichiometric equations method; the annual CO_2 emissions ranged between 3.7 x 10^6 tons for scenario 2 to 5.0 x 10^6 tons for scenario 1 for SNPS. For SSPS, the corresponding values were 3.0 x 10^6 for scenario 2 and 4.1 x 10^6 for scenario 1. As for the results obtained using the US-EPA emission factors, the annual CO_2 emissions ranged between 3.1 x 10^6 tons for SNPS and 2.5 x 10^6 tons for SSPS for scenario 2, while for scenario 1 these values were 4.5 x 10^6 tons for SNPS and 3.7 x 10^6 tons for SSPS. For

the Ecometrica (Kuwait specific), US-Average electricity emission factors, and the IEA Electricity emission factors (Kuwait specific), the annual CO_2 emissions for SNPS were 4.9 x 10^6, 4.3 x 10^6 and 6.1 x 10^6 tons, respectively, while for SSPS, they were 4.0 x 10^6, 3.5 x 10^6 and 5.0 x 10^6 tons, respectively. Finally, the IPCC calculations resulted in annual CO_2 emissions ranging between 3.3 x 10^6 tons for Scenario 2 to 5.1 x 10^6 tons for scenario 1 for SNPS and 2.7 x 10^6 tons for Scenario 2 to 4.2 x 10^6 tons for scenario 1 for SSPS, refer to Figure S4. Using the IPCC results as a base, Figure S5 shows the stoichiometric equations results had the smallest percentage error (i.e., average error = 5.00%) while those obtained using the US-EPA emission factors, the Ecometrica (Kuwait specific), the US-Average electricity emission factors, and the IEA Electricity emission factors (Kuwait specific) had average percentage errors of 8.62, 22.11, 7.10, and 51.36%, respectively.

Figure S6 shows the IPCC's results obtained for the above-listed two scenarios is addition to a third one reflecting the actual fuel consumed in 2016 as obtained by MEW. Obviously, a quick comparison between the emission values obtained for the three scenarios confirms the fact the units were not fully utilized throughout the year. The reader's attention is brought to the fact the vertical axis in Figure S6 is logarithmic in style, a choice made to allow the relatively negligible CH_4 and N_2O annual emissions to become visible on the chart. Figure S7 shows the results obtained for Zour South Power Station (ZSPS). Four scenarios were considered for this station:

1) the GTUs rely entirely on GO while the thermal units rely entirely on HFO,
2) the GTUs rely entirely on NG while the thermal units rely entirely on HFO,
3) the GTUs rely entirely on GO while the thermal units rely entirely on FO, and
4) the GTUs rely entirely on NG while the thermal units rely entirely on FO.

The calculated annual CO_2 emissions ranged between 26.2 x 10^6 tons for scenario 2 to 31.1 x 10^6 tons for scenario 3 for the stoichiometric equations calculation method. As for the results obtained using the US-EPA emission factors, the annual CO_2 emissions ranged between 23.5E06 tons for scenario 2 and 30.2 x 10^6 tons for scenario 3. For the calculation methods relying on Ecometrica (Kuwait specific) emission factors, US-Average electricity emission factors, and the IEA Electricity emission factors (Kuwait specific), the annual CO_2 emissions were 29.6 x 10^6, 26.0 x 10^6 and 36.7 x 10^6 tons, respectively, with no difference in the results of different scenarios. Finally, the IPCC calculations resulted in annual CO_2 emissions ranging between 25.4 x 10^6 tons for Scenario 2 and 31.4 x 10^6 tons for scenario 3, refer to Figure S7. Using the IPCC results as a base, Figure S8 shows the results obtained using the stoichiometric equations calculation method had the smallest percentage error (i.e., average error = 1.01%) while those obtained using the emission factors of US-EPA, the Ecometrica (Kuwait specific), the US-Average electricity sectors, and the IEA (Kuwait specific) had average percentage errors of 5.57, 5.30, 7.64, and 30.53%, respectively.

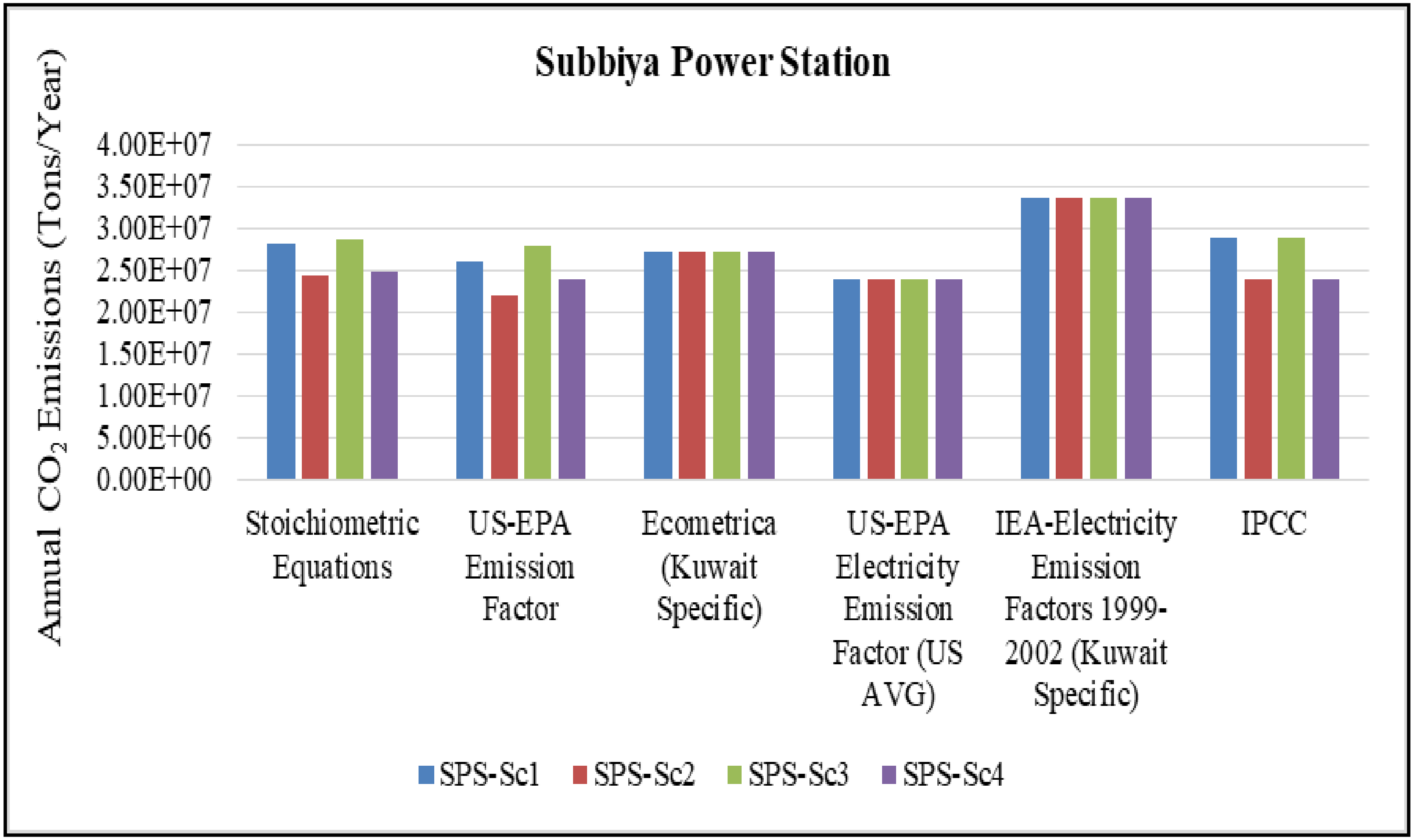

Figure S1. The annual CO_2 emissions for Subbiya Power Station based on the available possible scenarios using different calculation methods. Notes: US-EPA: United Stated Environment Protection Agency, IEA: International Energy Agency, IPCC: Intergovernmental Panel on Climate Change, SPS: Sabyia Power Station, Sc1: Scenario 1: GO + HFO, Sc2: Scenario 2: NG + HFO, Sc3: Scenario 3: GO + FO, Sc4: Scenario 4: NG + FO.

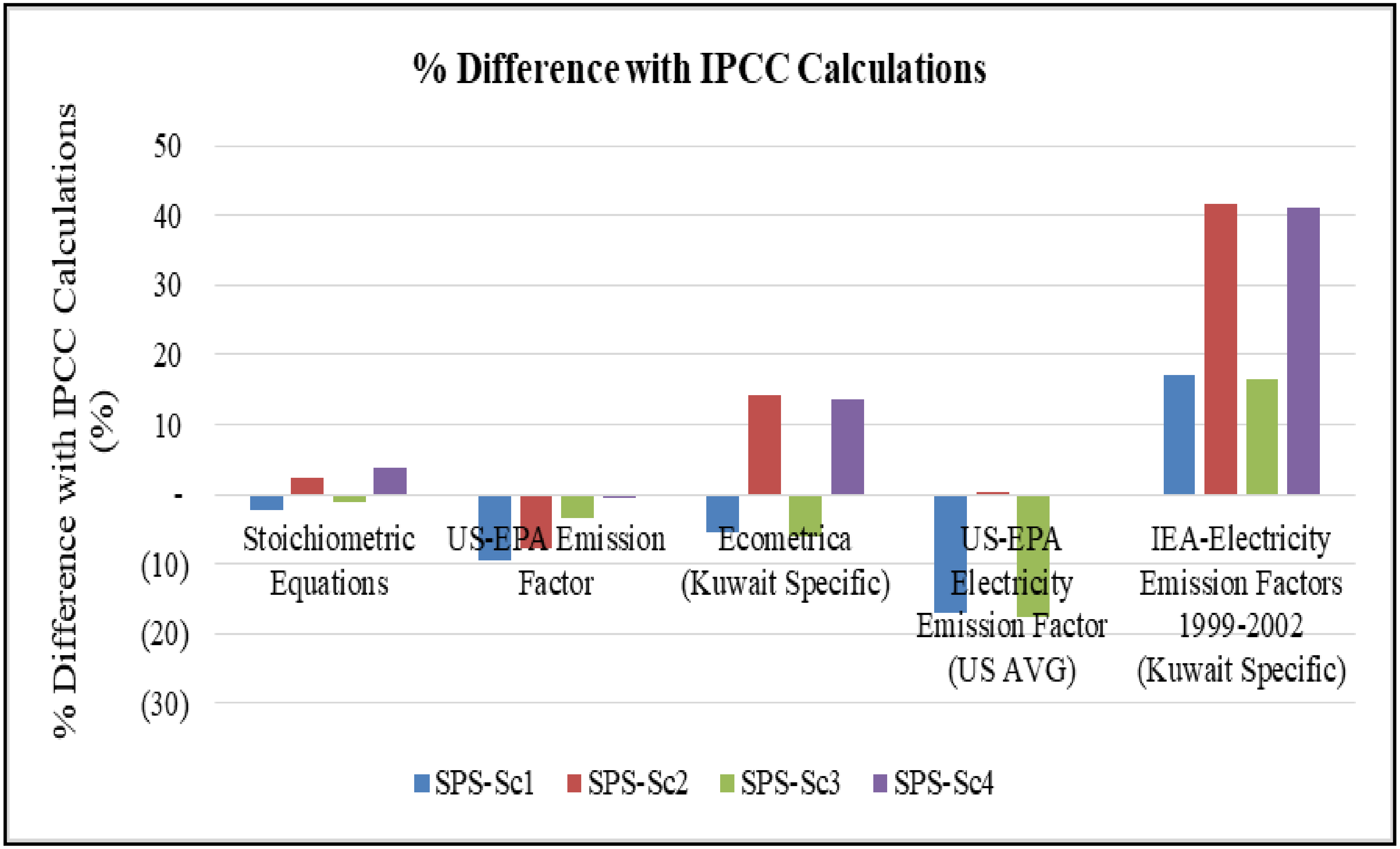

Figure S2. The percentage difference between the calculated annual CO_2 emissions for Sabyia Power Station using different calculation methods and that of IPCC.

Notes: US-EPA: United Stated Environment Protection Agency, IEA: International Energy Agency, IPCC: Intergovernmental Panel on Climate Change, SPS: Subbiya Power Station, Sc1: Scenario 1: GO + HFO, Sc2: Scenario 2: NG + HFO, Sc3: Scenario 3: GO + FO, Sc4: Scenario 4: NG + FO.

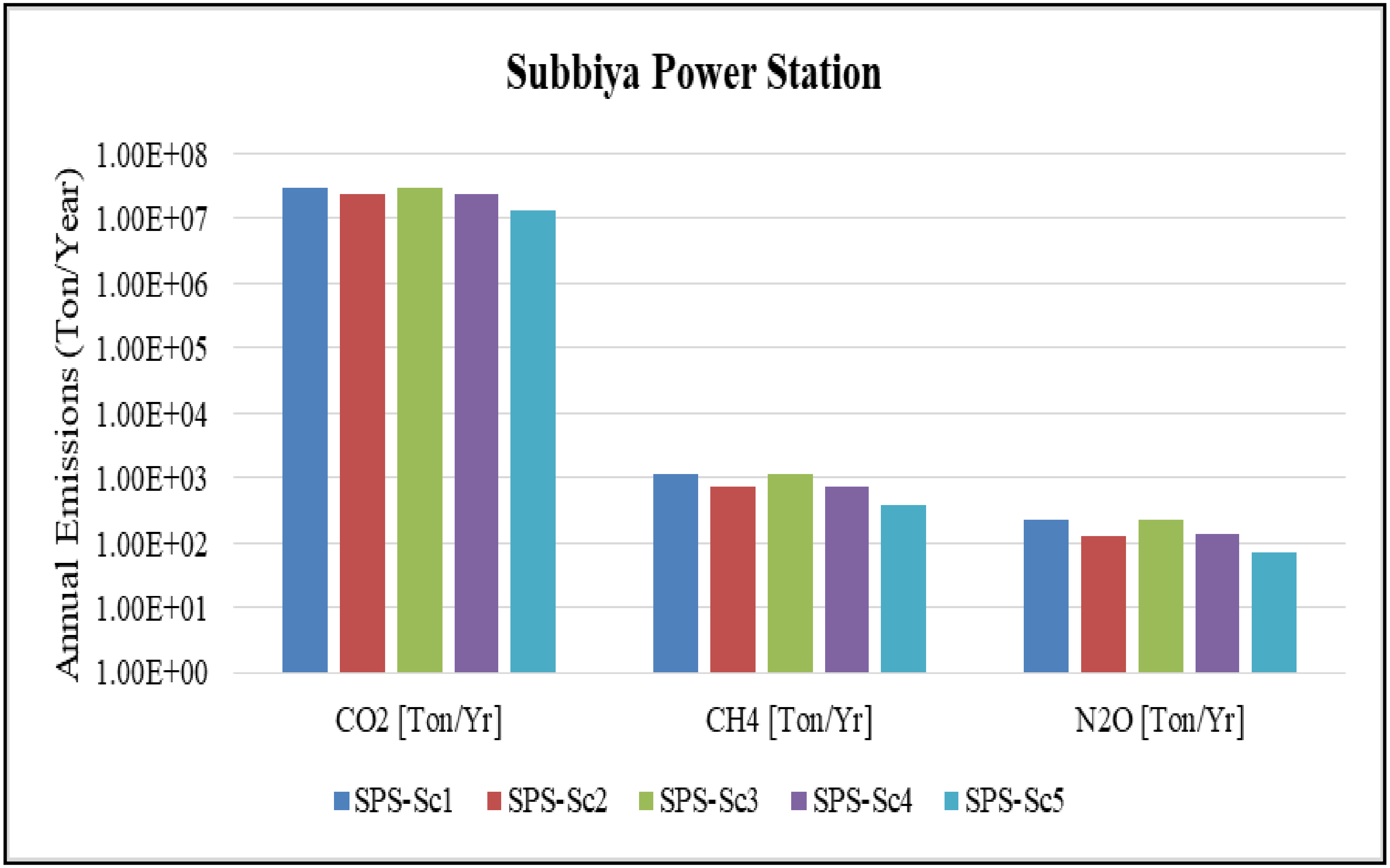

Figure S3. The annual CO_2, CH_4, and N_2O emissions for Subbiya Power Station based on the available possible scenarios using different calculation methods.

Notes: SPS: Subbiya Power Station, Sc1: Scenario 1: GO + HFO, Sc2: Scenario 2: NG + HFO, Sc3: Scenario 3: GO + FO, Sc4: Scenario 4: NG + FO, Sc5: Scenario 5: Actual Fuel Consumption.

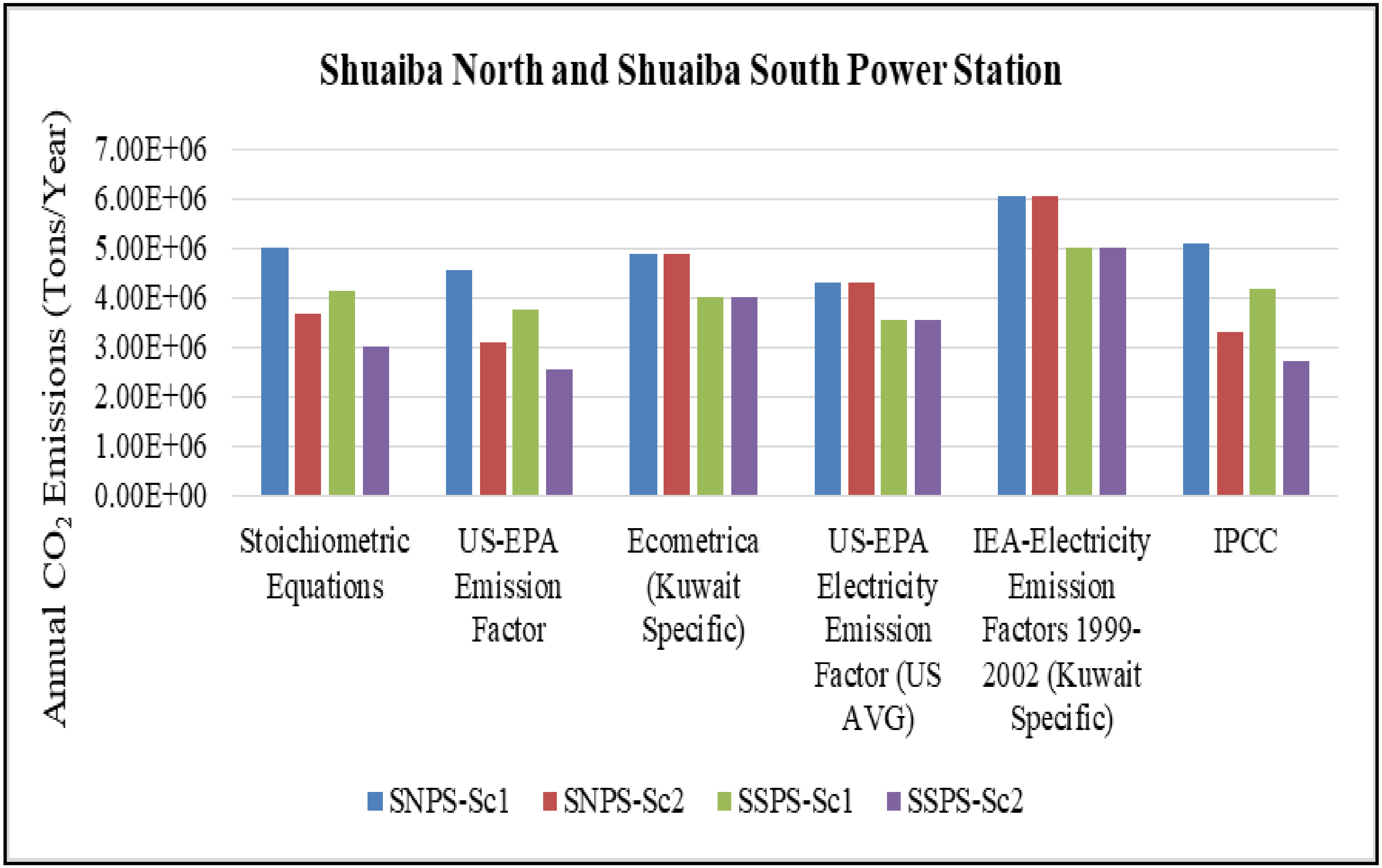

Figure S4. The annual CO_2 emissions for Shuaiba North and Shuaiba South Power Station based on the available possible scenarios using different calculation methods.

Notes: US-EPA: United Stated Environment Protection Agency, IEA: International Energy Agency, IPCC: Intergovernmental Panel on Climate Change, SNPS: Shuaiba North Power Station, SSPS: Shuaiba South Power Station, Sc1: Scenario 1: GO, Sc2: Scenario 2: NG.

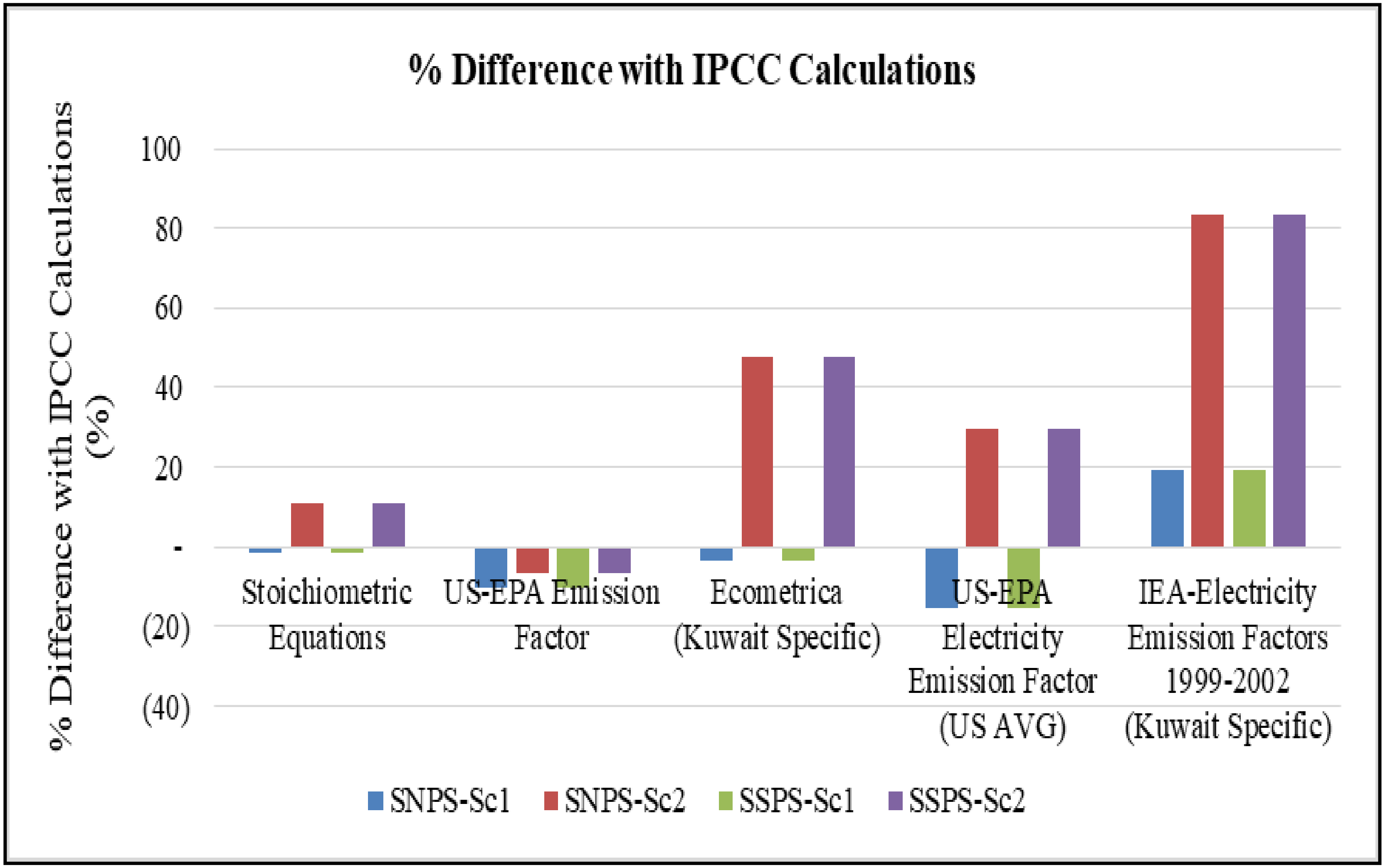

Figure S5. The percentage difference between the calculated annual CO_2 emissions for Shuaiba North and Shuaiba South Power Station using different calculation methods and that of IPCC.

Notes: US-EPA: United Stated Environment Protection Agency, IEA: International Energy Agency, IPCC: Intergovernmental Panel on Climate Change, SNPS: Shuaiba North Power Station, SSPS: Shuaiba South Power Station, Sc1: Scenario 1: GO, Sc2: Scenario 2: NG.

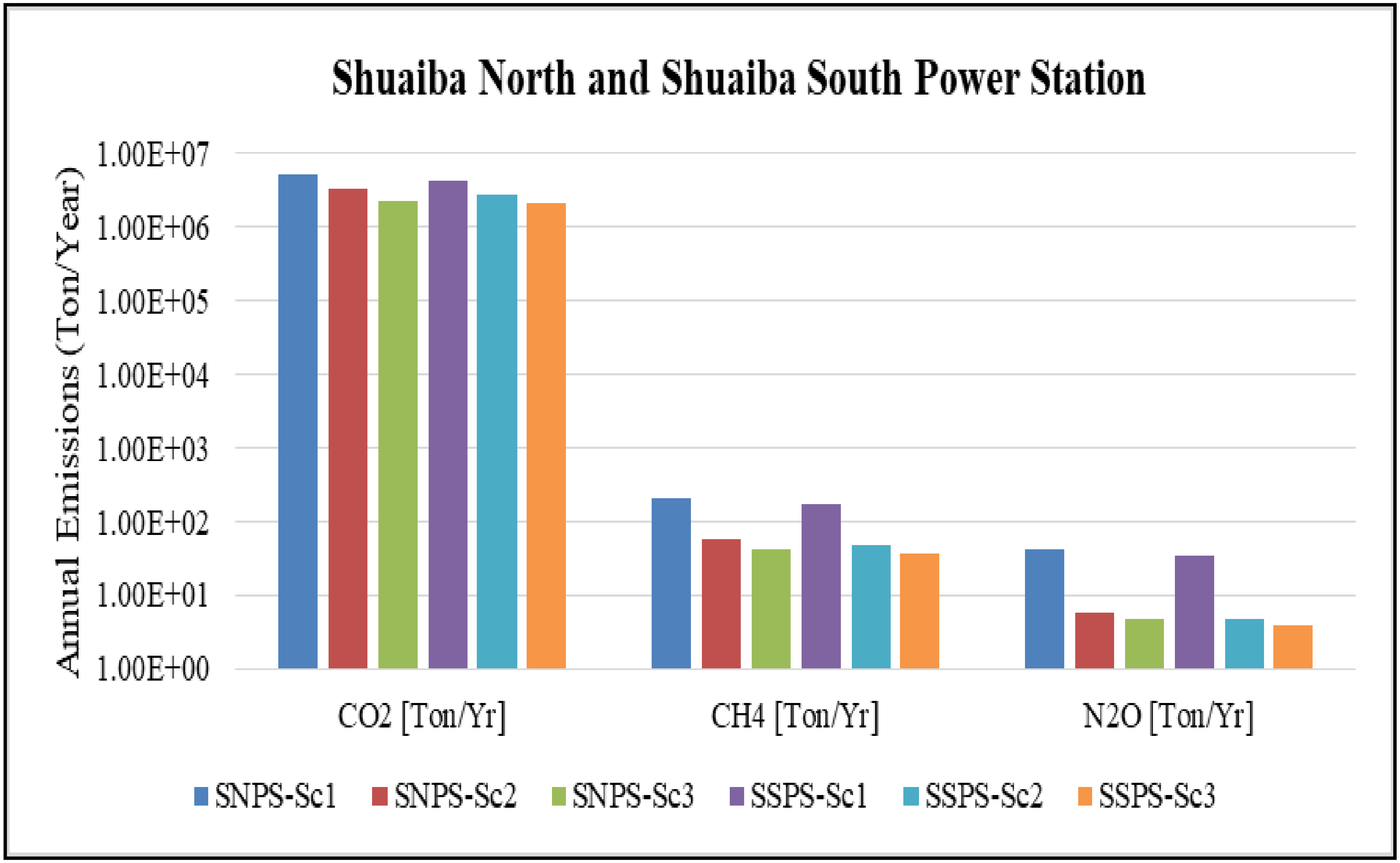

Figure S6. The annual CO_2, CH_4, and N_2O emissions for Shuaiba North and Shuaiba Power Station based on the available possible scenarios using different calculation methods.

Notes: SNPS: Shuaiba North Power Station, SSPS: Shuaiba South Power Station, Sc1: Scenario 1: GO, Sc2: Scenario 2: NG, Sc3: Scenario 3: Actual Fuel Consumption.

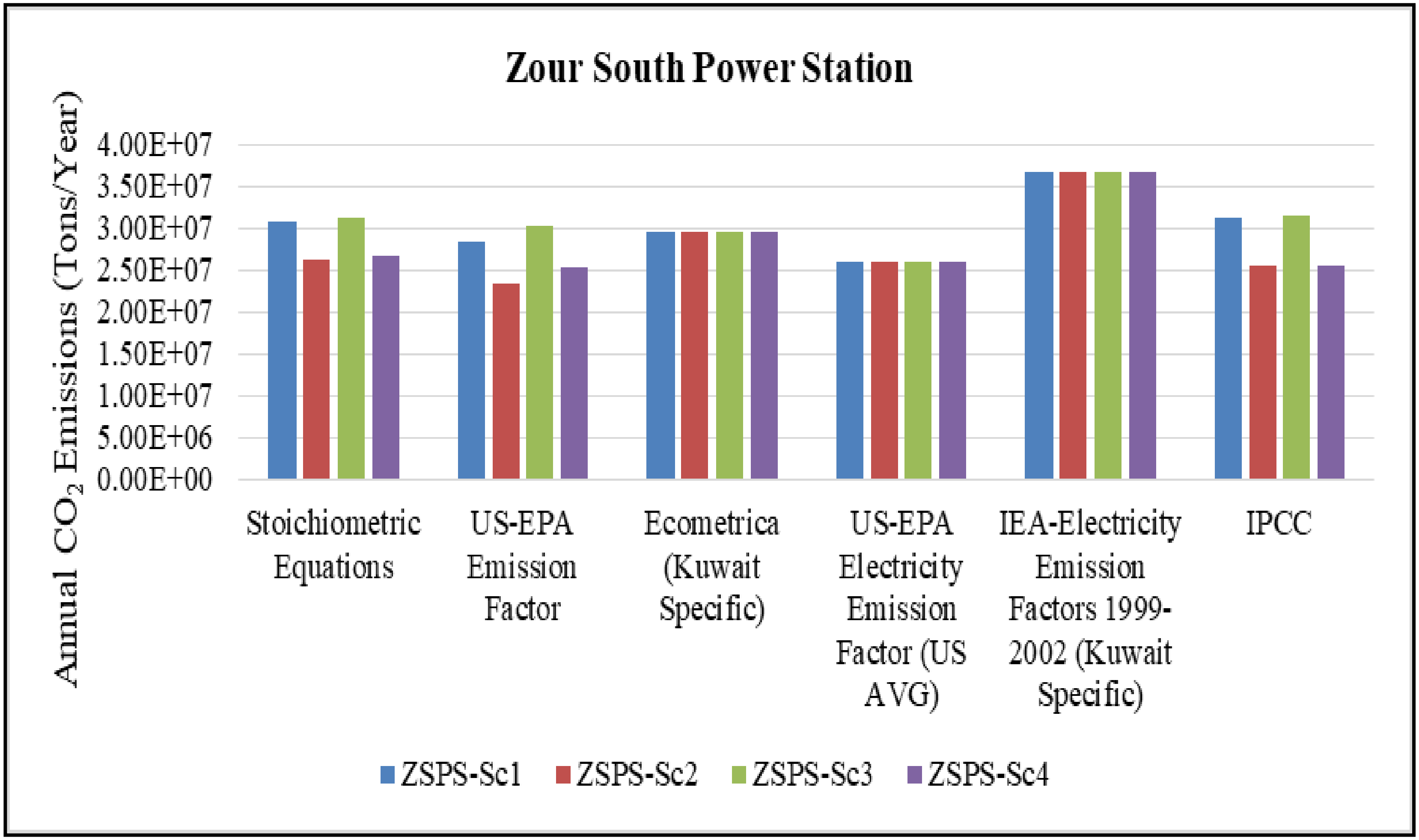

Figure S7. The annual CO_2 emissions for Zour South Power Station based on the available possible scenarios using different calculation methods. Notes: US-EPA: United Stated Environment Protection Agency, IEA: International Energy Agency, IPCC: Intergovernmental Panel on Climate Change, ZSPS: Zour South Power Station, Sc1: Scenario 1: GO + HFO, Sc2: Scenario 2: NG + HFO, Sc3: Scenario 3: GO + FO, Sc4: Scenario 4: NG + FO.

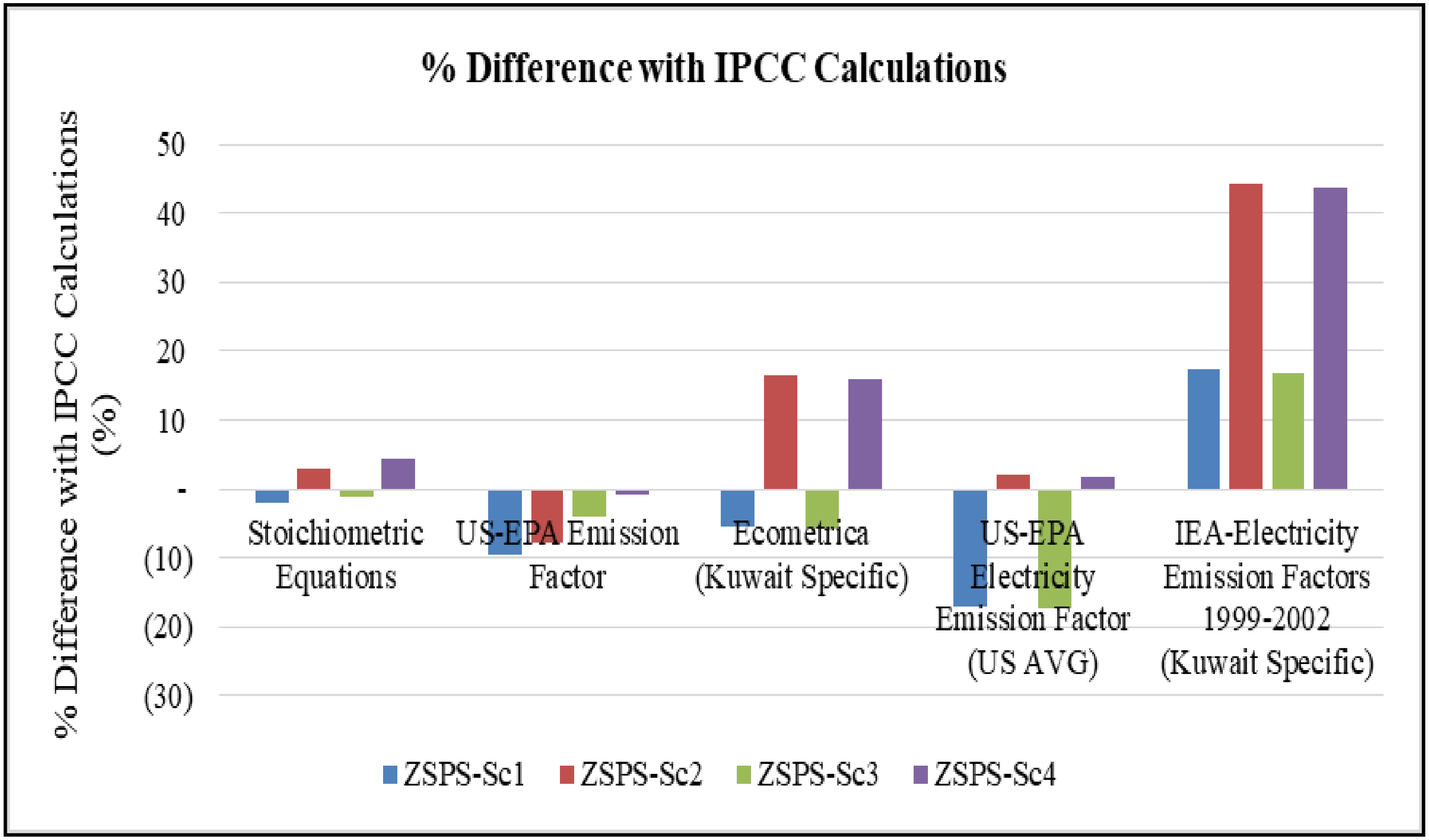

Figure S8. The percentage difference between the calculated annual CO_2 emissions for Zour South Power Station using different calculation methods and that of IPCC.

Notes: US-EPA: United Stated Environment Protection Agency, IEA: International Energy Agency, IPCC: Intergovernmental Panel on Climate Change, ZSPS: Zour South Power Station, Sc1: Scenario 1: GO + HFO, Sc2: Scenario 2: NG + HFO, Sc3: Scenario 3: GO + FO, Sc4: Scenario 4: NG + FO.

Figure S9 shows the IPCC's results obtained for the above-listed four scenarios is addition to a fifth scenario reflecting the actual fuel consumed in 2016 as obtained by MEW. Obviously, a quick comparison between the emission values obtained for the five scenarios shows the emission rates for the 5th scenario as lowest confirming by which the fact the ZSPS units were not fully utilized throughout the year. The reader's attention is brought to the fact the vertical axis in Figure S9 is logarithmic in style, a choice made to allow the relatively negligible CH_4 and N_2O annual emissions to become visible on the chart. One more series, i.e. that for Zour North Power Station (ZNPS), was added to this chart. The units at ZNPS do not belong to MEW, still, their GHGs emissions were added to illustrate the lower contribution of these units to GHGs emissions. Figure S10 shows the results obtained for Doha West Power Station (DWPS). Four scenarios were considered for this station:

- the GTUs rely entirely on GO while the thermal units rely entirely on HFO,
- the GTUs rely entirely on NG while the thermal units rely entirely on HFO,
- the GTUs rely entirely on GO while the thermal units rely entirely on FO, and
- the GTUs rely entirely on NG while the thermal units rely entirely on FO.

The calculated annual CO_2 emissions ranged between 14.6 x 10^6 tons for scenario 2 to 15.3 x 10^6 tons for scenario 3 for the stoichiometric equations calculation method. As for the results obtained using the US-EPA emission factors, the annual CO_2 emissions ranged between 13.7E06 tons for scenario 2 to 15.8 x 10^6 tons for scenario 3. When the emission factors of the Ecometrica (Kuwait specific), US-Average for electricity sector, and the IEA Electricity (Kuwait specific) were used, the annual CO_2 emissions were 14.2 x 10^6, 12.4 x 10^6 and 17.6 x 10^6 tons, respectively, with no difference in the results of different scenarios. Finally, the IPCC calculations resulted in annual CO_2 emissions ranging between 15.0 x 10^6 tons for Scenario 2 and 15.4 x 10^6 tons for scenario 3, refer to Figure S10. Using the IPCC results as a base, Figure S11 shows the results obtained using the stoichiometric equations calculation method had the smallest percentage error (i.e., average error = 1.83%) while those obtained using the emission factors of US-EPA, the Ecometrica (Kuwait specific), the US-Average electricity sectors, and the IEA (Kuwait specific) had average percentage errors of 2.83, 6.69, 18.16, and 15.67%, respectively.

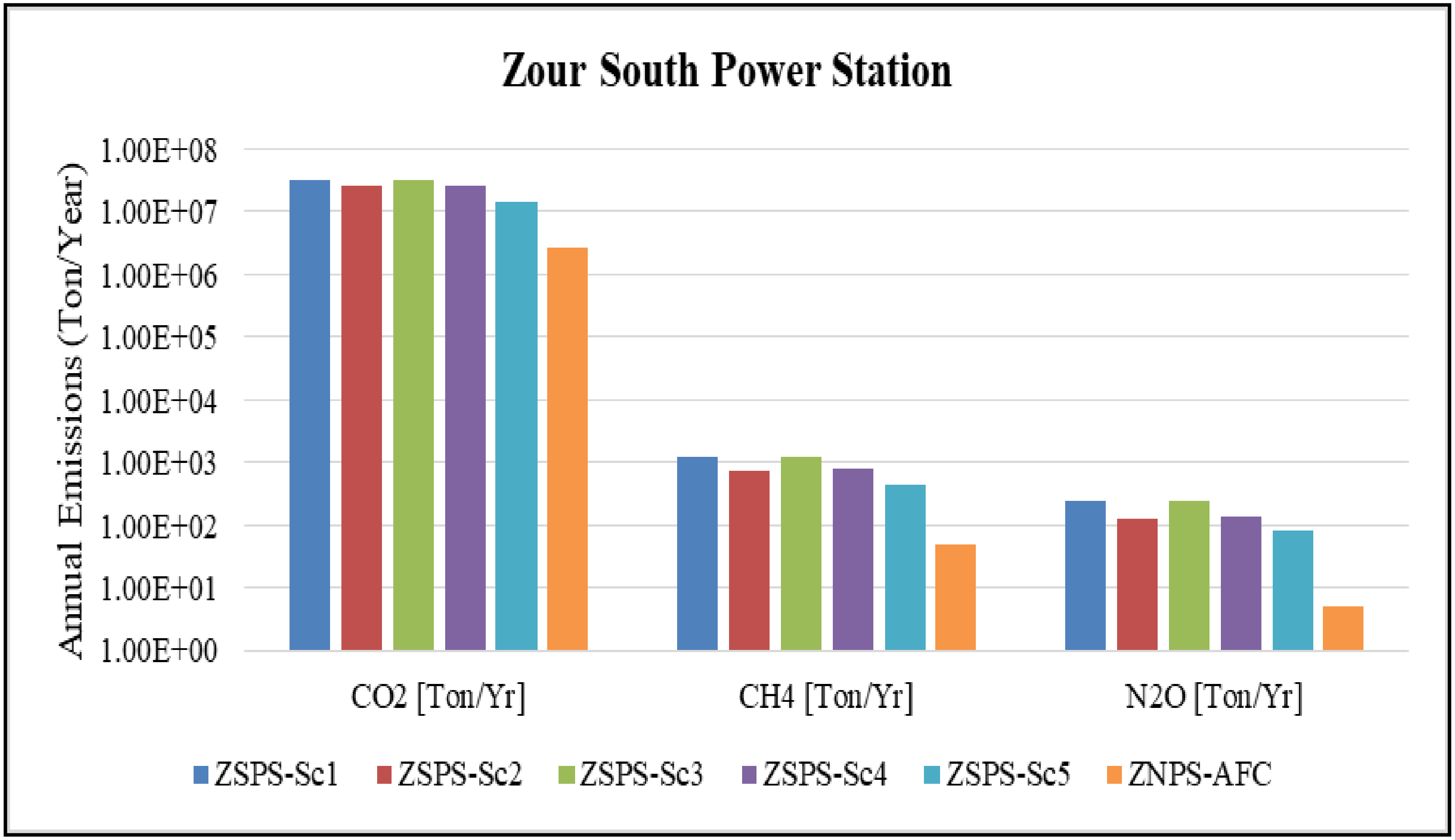

Figure S9. The annual CO_2, CH_4, and N_2O emissions for Zour South Power Station based on the available possible scenarios using different calculation methods.

Notes: US-EPA: United Stated Environment Protection Agency, IEA: International Energy Agency, IPCC: Intergovernmental Panel on Climate Change, ZSPS: Zour South Power Station, ZNPS: Zour North Power Station, Sc1: Scenario 1: GO + HFO, Sc2: Scenario 2: NG + HFO, Sc3: Scenario 3: GO + FO, Sc4: Scenario 4: NG + FO, Scenario 5: Actual Fuel Consumption, AFC: Actual Fuel Consumption.

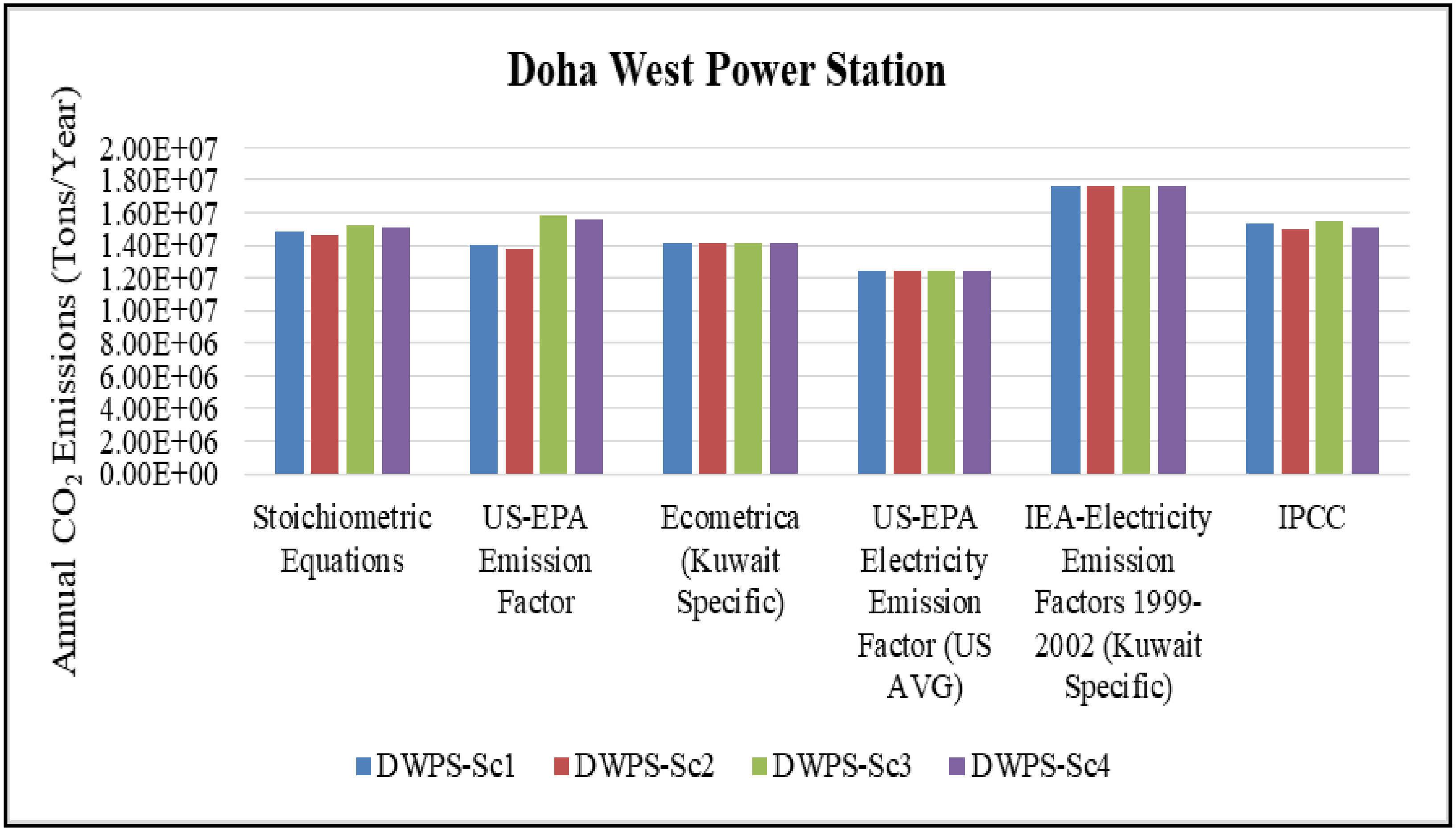

Figure S10. The annual CO_2 emissions for Doha West Power Station based on the available possible scenarios using different calculation methods. Notes: US-EPA: United Stated Environment Protection Agency, IEA: International Energy Agency, IPCC: Intergovernmental Panel on Climate Change, DWPS: Doha West Power Station, Sc1: Scenario 1: GO + HFO, Sc2: Scenario 2: NG + HFO, Sc3: Scenario 3: GO + FO, Sc4: Scenario 4: NG + FO.

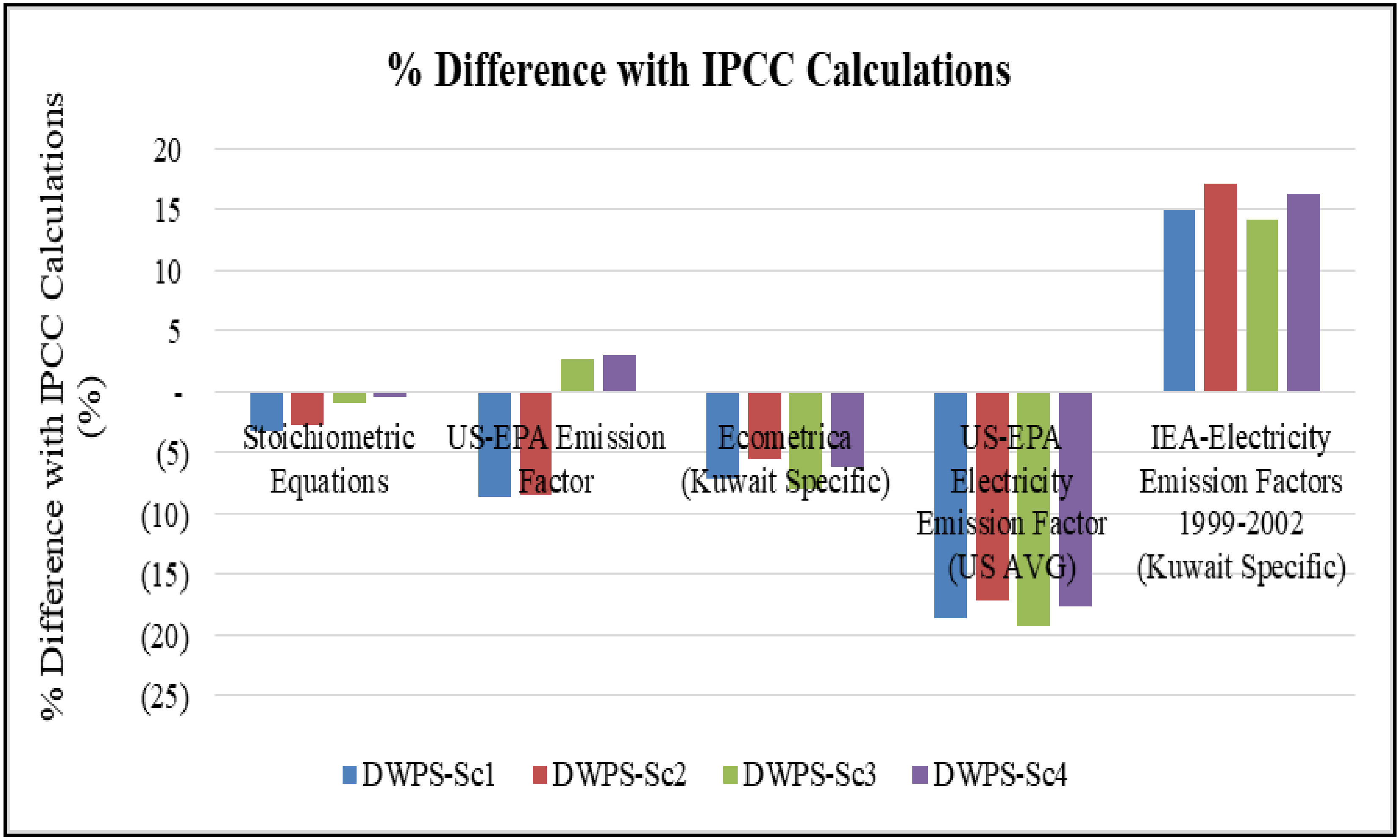

Figure S11. The percentage difference between the calculated annual CO_2 emissions for Doha West Power Station using different calculation methods and that of IPCC.

Notes: US-EPA: United Stated Environment Protection Agency, IEA: International Energy Agency, IPCC: Intergovernmental Panel on Climate Change, DWPS: Doha West Power Station, Sc1: Scenario 1: GO + HFO, Sc2: Scenario 2: NG + HFO, Sc3: Scenario 3: GO + FO, Sc4: Scenario 4: NG + FO.

Figure S12 shows the IPCC's results obtained for the above-listed four scenarios is addition to a fifth scenario reflecting the actual fuel consumed in 2016 as obtained by MEW. Obviously, a quick comparison between the emission values obtained for the five scenarios shows the emission rates for the 5th scenario as lowest confirming by which the fact the DWPS units were not fully utilized throughout the year. The reader's attention is brought to the fact the vertical axis in Figure S12 is logarithmic in style, a choice made to allow the relatively negligible CH_4 and N_2O annual emissions to become visible on the chart. Figure S13 shows the results obtained for Doha East Power Station (DEPS). Four scenarios were considered for this station:

- the GTUs rely entirely on GO while the thermal units rely entirely on HFO,
- the GTUs rely entirely on NG while the thermal units rely entirely on HFO,
- the GTUs rely entirely on GO while the thermal units rely entirely on FO, and
- the GTUs rely entirely on NG while the thermal units rely entirely on FO.

The calculated annual CO_2 emissions ranged between 6.6 x 10^6 tons for scenario 2 to 6.9 x 10^6 tons for scenario 3 for the stoichiometric equations calculation method. As for the results obtained using the US-EPA emission factors, the annual CO_2 emissions ranged between 6.2E06 tons for scenario 2 and 7.2 x 10^6 tons for scenario 3. For the calculation methods relying on Ecometrica (Kuwait specific) emission factors, US-Average electricity emission factors, and the IEA Electricity emission factors (Kuwait specific), the annual CO_2 emissions were 6.5 x 10^6, 5.7 x 10^6 and 8.0 x 10^6 tons, respectively, with no difference in the results of different scenarios. Finally, the IPCC calculations resulted in annual CO_2 emissions ranging between 6.7 x 10^6 tons for Scenario 2 and 7.0 x 10^6 tons for scenario 3, refer to Figure S14. Using the IPCC results as a base, Figure S14 shows the results obtained using the stoichiometric equations calculation method had the smallest percentage error (i.e., average error = 1.65%) while those obtained using the emission factors of US-EPA, the Ecometrica (Kuwait specific), the US-Average electricity sectors, and the IEA (Kuwait specific) had average percentage errors of 3.03, 5.92, 17.48 and 16.62%, respectively.

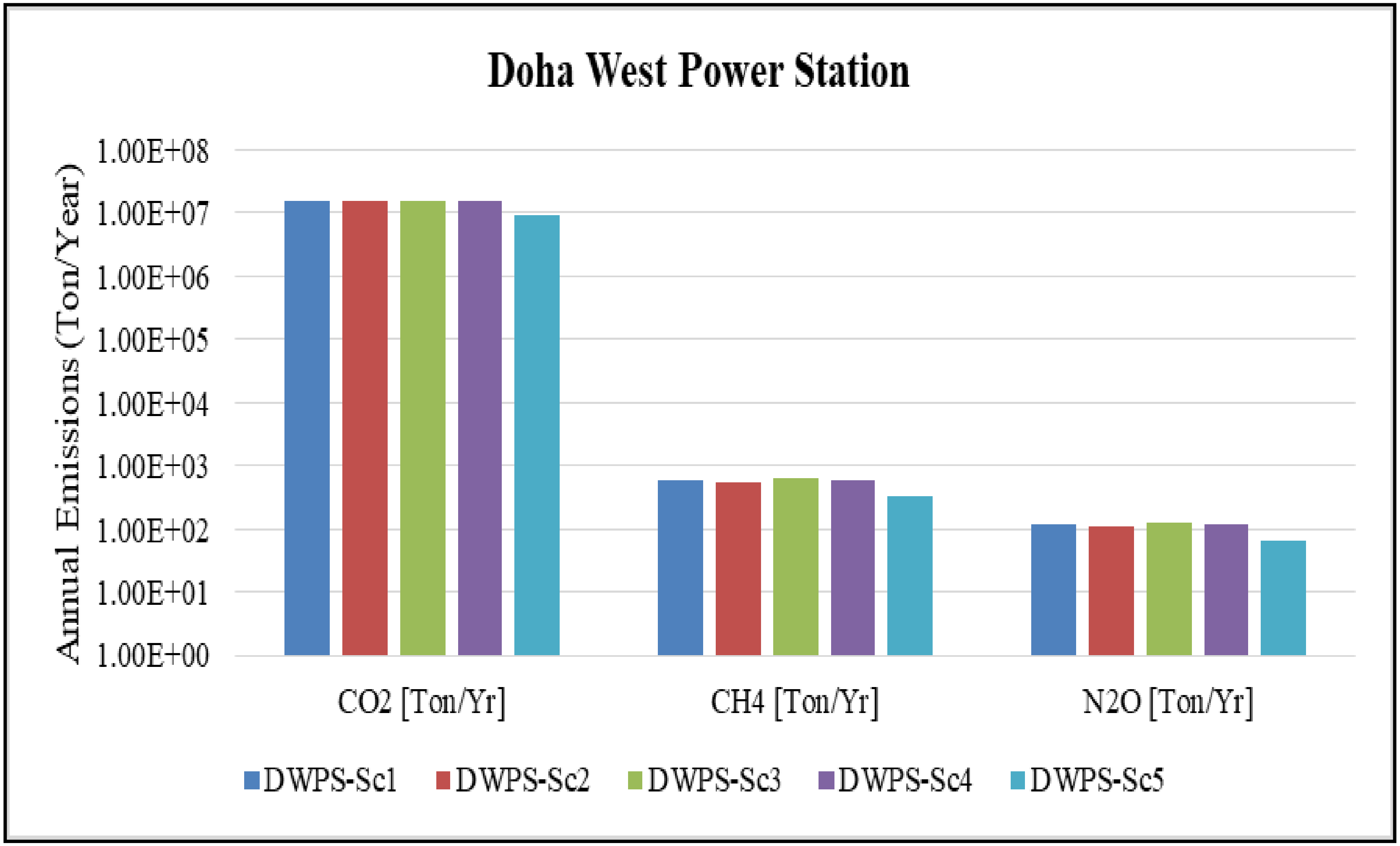

Figure S12 The annual CO_2, CH_4, and N_2O emissions for Doha West Power Station based on the available possible scenarios using different calculation methods.

Notes: DWPS: Doha West Power Station, Sc1: Scenario 1: GO + HFO, Sc2: Scenario 2: NG + HFO, Sc3: Scenario 3: GO + FO, Sc4: Scenario 4: NG + FO, Sc5: Scenario 5: Actual Fuel Consumption.

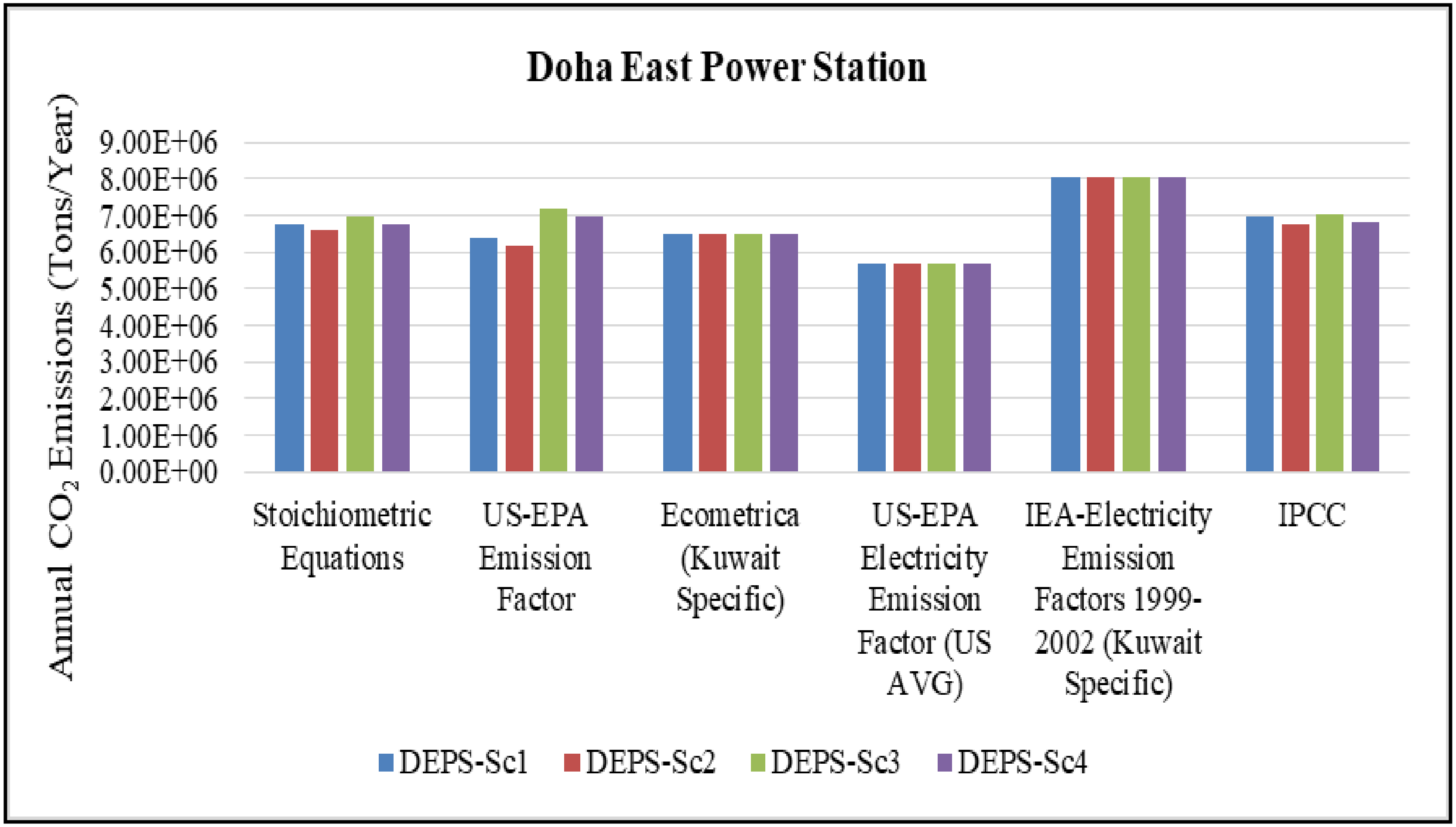

Figure S13 The annual CO_2 emissions for Doha East Power Station based on the available possible scenarios using different calculation methods.

Notes: US-EPA: United Stated Environment Protection Agency, IEA: International Energy Agency, IPCC: Intergovernmental Panel on Climate Change, DEPS: Doha East Power Station, Sc1: Scenario 1: GO + HFO, Sc2: Scenario 2: NG + HFO, Sc3: Scenario 3: GO + FO, Sc4: Scenario 4: NG + FO.

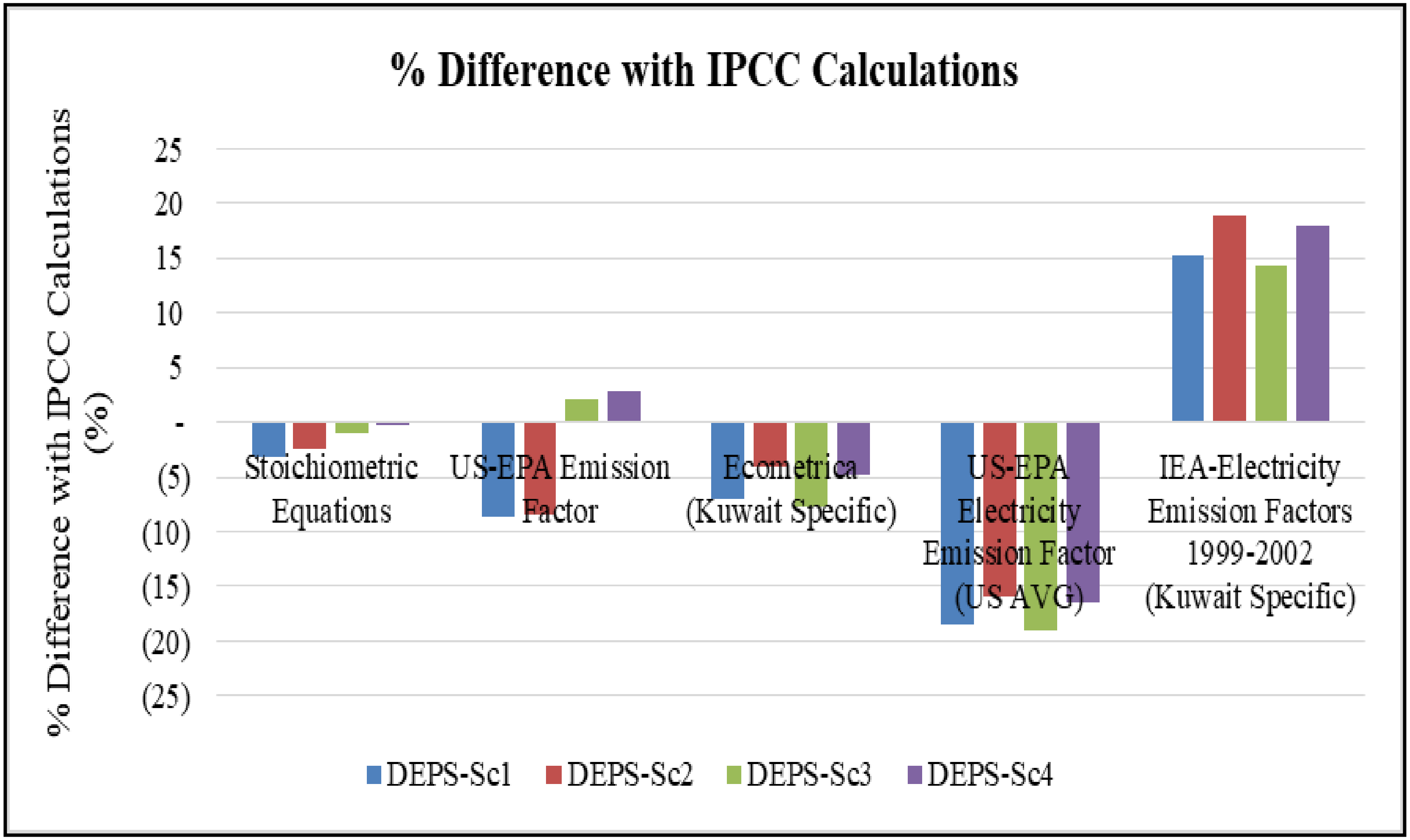

Figure S14. The percentage difference between the calculated annual CO_2 emissions for Doha East Power Station using different calculation methods and that of IPCC.

Notes: US-EPA: United Stated Environment Protection Agency, IEA: International Energy Agency, IPCC: Intergovernmental Panel on Climate Change, DEPS: Doha East Power Station, Sc1: Scenario 1: GO + HFO, Sc2: Scenario 2: NG + HFO, Sc3: Scenario 3: GO + FO, Sc4: Scenario 4: NG + FO.

Figure S15 shows the IPCC's results obtained for the above-listed four scenarios is addition to a fifth scenario reflecting the actual fuel consumed in 2016 as obtained by MEW. Obviously, a quick comparison between the emission values obtained for the five scenarios confirms the fact the units were not fully utilized throughout the year. The reader's attention is brought to the fact the vertical axis in Figure S15 is logarithmic in style, a choice made to allow the relatively negligible CH_4 and N_2O annual emissions to become visible on the chart. Figure S16 shows the results obtained for Shuwaikh Desalination Plant (SHDP). Two scenarios were considered for each station:

- the GTUs rely entirely on GO, and
- the GTUs rely entirely on NG.

For the stoichiometric equations method; the annual CO_2 emissions ranged between 1.4 x 10^6 tons for scenario 1 to 1.5 x 10^6 tons for scenario 2. As for the results obtained using the US-EPA emission factors, the annual CO_2 emissions ranged between 0.9 x 10^6 tons for scenario 2 and 1.3 x 10^6 tons for scenario 1. For the Ecometrica (Kuwait specific), US-Average electricity emission factors, and the IEA Electricity emission factors (Kuwait specific), the annual CO_2 emissions were 1.4 x 10^6, 1.2 x 10^6 and 1.7 x 10^6 tons, respectively. Finally, the IPCC calculations resulted in annual CO_2 emissions ranging between 1.0E06 tons for Scenario 2 and 1.5 x 10^6 tons for scenario 1, refer to Figure S16. Figure S17 depicts the percentage error associated with each calculation method assuming the IPCC one was the reference. Figure S18 shows the IPCC's results obtained for the above-listed two scenarios is addition to a third one reflecting the actual fuel consumed in 2016 as obtained by MEW. Obviously, a quick comparison between the emission values obtained for the three scenarios confirms the fact the units were not fully utilized throughout the year. The reader's attention is brought to the fact the vertical axis in Figure S18 is logarithmic in style, a choice made to allow the relatively negligible CH_4 and N_2O annual emissions to become visible on the chart.

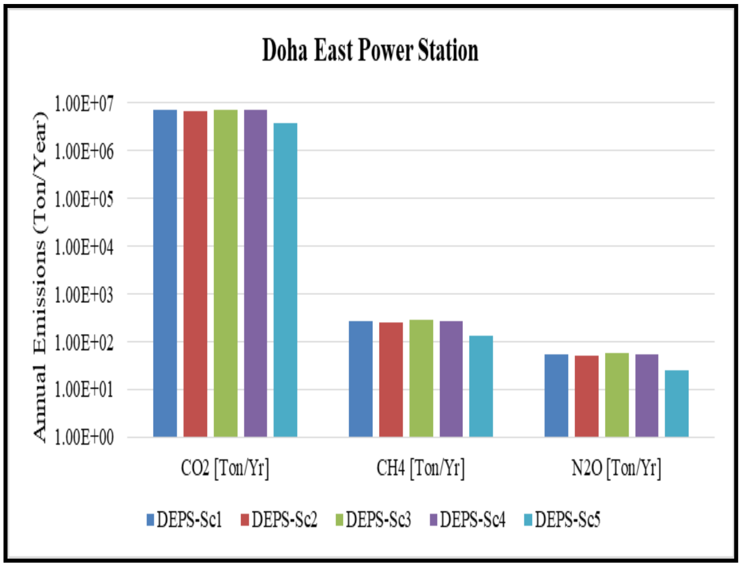

Figure S15. The annual CO_2, CH_4, and N_2O emissions for Doha East Power Station based on the available possible scenarios using different calculation methods.

Notes: DEPS: Doha East Power Station, Sc1: Scenario 1: GO + HFO, Sc2: Scenario 2: NG + HFO, Sc3: Scenario 3: GO + FO, Sc4: Scenario 4: NG + FO, Sc5: Scenario 5: Actual Fuel Consumption.

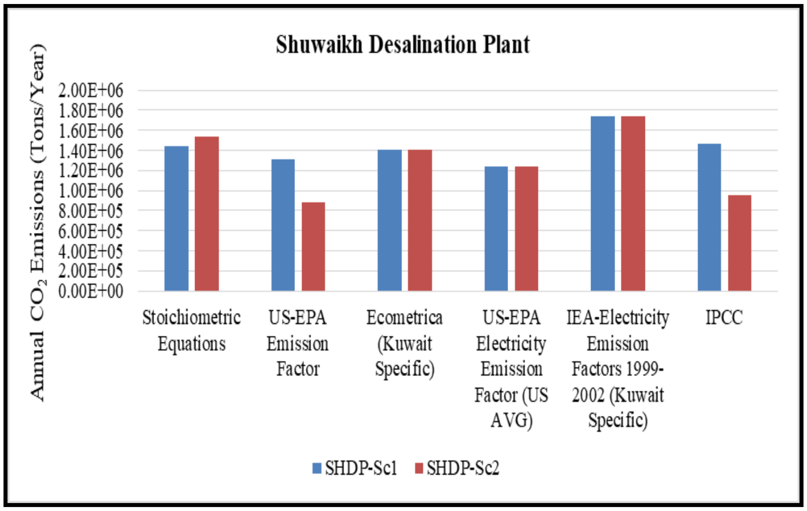

Figure S16. The annual CO_2 emissions for Shuwaikh Desalination Plant based on the available possible scenarios using different calculation methods.

Notes: US-EPA: United Stated Environment Protection Agency, IEA: International Energy Agency, IPCC: Intergovernmental Panel on Climate Change, SHDP: Shuwaikh Desalination Plant, Sc1: Scenario 1: GO, Sc2: Scenario 2: NG.

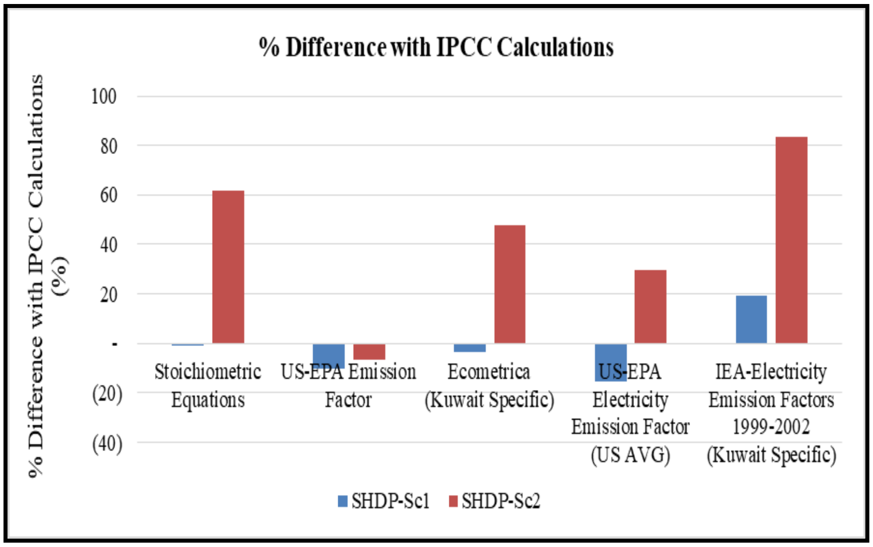

Figure S17. The percentage difference between the calculated annual CO_2 emissions for Shuwaikh Desalination Plant using different calculation methods and that of IPCC.

Notes: US-EPA: United Stated Environment Protection Agency, IEA: International Energy Agency, IPCC: Intergovernmental Panel on Climate Change, SHDP: Shuwaikh Desalination Plant, Sc1: Scenario 1: GO, Sc2: Scenario 2: NG.

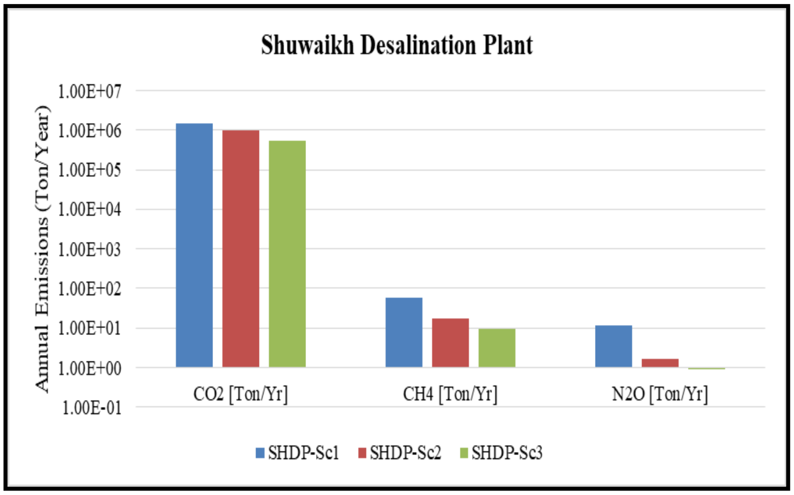

Figure S18. The annual CO_2, CH_4, and N_2O emissions for Shuwaikh Desalination Plant based on the available possible scenarios using different calculation methods.

Notes: US-EPA: United Stated Environment Protection Agency, IEA: International Energy Agency, IPCC: Intergovernmental Panel on Climate Change, SHDP: Shuwaikh Desalination Plant, Sc1: Scenario 1: GO, Sc2: Scenario 2: NG, Sc3: Scenario 3: Actual Fuel Consumption.
